# Signal Transducer and Activator of Transcription (STATs) Proteins in Cancer and Inflammation: Functions and Therapeutic Implication

**DOI:** 10.3389/fonc.2019.00048

**Published:** 2019-02-21

**Authors:** Chin-Yap Loh, Aditya Arya, Ahmed Fadhil Naema, Won Fen Wong, Gautam Sethi, Chung Yeng Looi

**Affiliations:** ^1^School of Biosciences, Faculty of Health and Medical Sciences, Taylor's University, Subang Jaya, Malaysia; ^2^School of Medicine, Faculty of Health and Medical Sciences, Taylor's University, Subang Jaya, Malaysia; ^3^Center of Biotechnology Researches, University of Al-Nahrain, Baghdad, Iraq; ^4^Department of Medical Microbiology, Faculty of Medicine, University of Malaya, Kuala Lumpur, Malaysia; ^5^Department of Pharmacology, Yong Loo Lin School of Medicine, National University of Singapore, Singapore, Singapore

**Keywords:** STAT transcription factors, cancer, inflammation, therapeutic implication, STAT3

## Abstract

Signal Transducer and Activator of Transcription (STAT) pathway is connected upstream with Janus kinases (JAK) family protein and capable of integrating inputs from different signaling pathways. Each family member plays unique functions in signal transduction and crucial in mediating cellular responses to different kind of cytokines. STAT family members notably STAT3 and STAT5 have been involved in cancer progression whereas STAT1 plays opposite role by suppressing tumor growth. Persistent STAT3/5 activation is known to promote chronic inflammation, which increases susceptibility of healthy cells to carcinogenesis. Here, we review the role of STATs in cancers and inflammation while discussing current therapeutic implications in different cancers and test models, especially the delivery of STAT3/5 targeting siRNA using nanoparticulate delivery system.

## Introduction: The Role of STAT Family Members in Cancer and Inflammation

The first direct link between STAT family proteins and carcinoma in human derived from research works that demonstrate that constitutively activated STAT3 is crucial for the carcinogenesis of head and neck cancer and multiple myeloma cells (1, 2). There is evidence indicated that antagonizing STAT3 signaling could induce cell death in human U266 myeloma cells (1). Subsequently, several types of solid tumors, leukemia, and lymphomas have been linked with constitutive activation of STAT3. IL-6 autocrine or paracrine loops was recognized as source for composition of STAT3 activity in myeloma and prostate malignant cell lines (3). Transforming growth factor-α (TGF-α)-mediated epidermal growth factor receptor (EGFR) signaling plays a vital role for the activation of STAT3 in some head and neck cancer cell lines (2). Hepatocyte growth factor (HGF) signaling via the receptor, c-MET, is related with the transformation of leiomyosarcoma cells, breast carcinoma cells, melanoma cells, and lung cancer cells in conjunction with SRC kinase which stimulate the expression of STAT3 (4–7).

Evidences have shown that constitutive activation of STAT5 are the leading causes of tumorigenesis (8). STAT proteins are indispensable in coordinating the response of hematopoietic cells to a wide range of cytokines. In fact, STAT5 activation is crucial for cancer progression in chronic myelogenous leukemia (CML) and myeloproliferative disease induced by TEL–JAK2 (9, 10). Aberrant chromosomal translocation BCR–ABL kinase and activation of FMS-like tyrosine kinase 3 (FLT3) receptor tyrosine can induce STAT 5 activation in CML and Acute myeloid leukemia (AML), respectively. However, not all family members in STAT proteins are promoting cancer progression. For instance, activated STAT1 appears to exert pro-apoptotic and anti-proliferative effect as STAT1-null mice lead to higher risk of tumor development than controls (11, 12), these results indicate that STAT1 has the tumor-suppressing properties like TP53 and could be an antagonist for STAT3 and STAT5 activation. All these studies showed that STAT1 unlikely to promote tumor cell growth in human.

Abnormally high activity of the Nuclear Factor Kappa B (NF-κB) pathway and Cyclooxygenase-2 (COX-2) activity induced by inflammatory mediators or reactive nitrogen oxygen species (RNOS) might aid inflammation-mediated tumorigenesis. STAT3 has close association with inflammation which is subsequently linked with tumor initiation due to mutation in genetic makeup of malignant cells (13, 14), in addition to this different environmental influences such as, stress, carcinogenic agents, smoking and radiations (15, 16). Apart from that, STAT proteins are crucial mediators of immunity against pathogens, and in the advancement of inflammatory disorder (17). Emphatically, initial natural protective event which is connected with STATs was inflammation in which STAT1 shows anti-viral activity while STAT4 and STAT6 involves in polarization of T helper cells. IFN/STAT1 pathway can induce inflammation in several ways, for example increasing the production of chemokines, managing the differentiation and apoptosis of hematopoietic cells and initiating formation of reactive oxygen species and nitric oxide (18). STAT2, STAT4, and STAT6 are stimulated by certain cytokines, such as IL-12, IL-4/IL-13, and IFN-α (19). Development of T helper cells by STAT6 contributes in a positive way in modulating inflammation during allergy while in negative way in autoimmunity (20). Consequently, differentiation of type 1 T helper cells by STAT4 was essential for autoimmune and inflammatory disorders. This model has highlighting the fact that numerous STAT proteins can participate in differentiation of single T helper phenotype, and single STAT protein can be essential for the expansion of numerous T helper subsets (21, 22).

As discussed above, STAT1 protein mainly function as tumor suppressor, suggesting that not all STAT proteins participate in the progression of inflammation and malignancy in human. Since STAT3/5 is directly implicated in oncogenesis and inflammation, the following sections will focus more on their molecular regulation and therapeutic implications of these proteins.

## Molecular Regulation of STAT 3/5 Protein Activation

**Figure 1:**
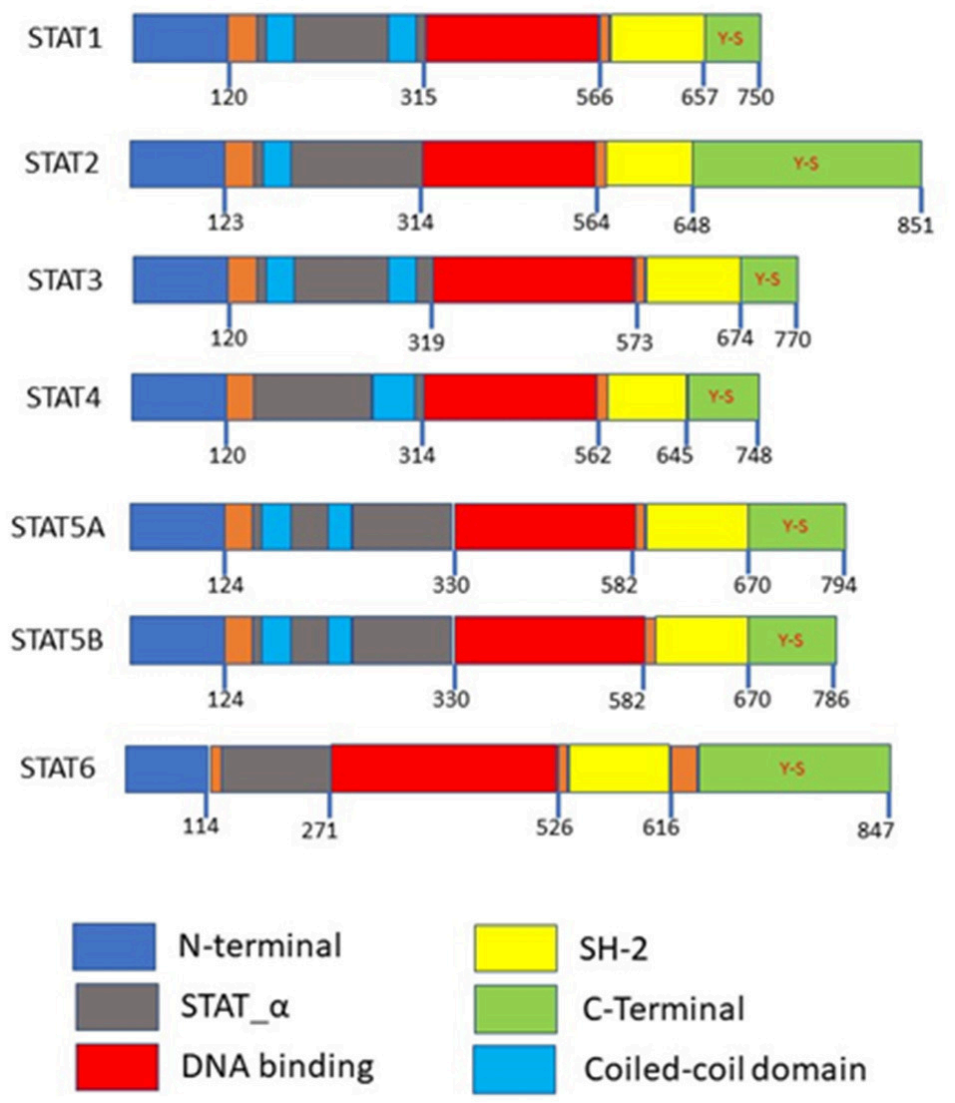
Domain structures of the STAT family members. N-terminal is required for protein interactions, SH-2 domains are required for dimerization, both the N-terminal and SH-2 domains mediated homo or heterodimer formation. Coiled-coil domains are required as a nuclear localization signal for activation. Y-S within the C-terminal are the phosphorylation sites.

Accumulating research has shown that how STAT3 constitutively activated in numerous types of cancer, including multiple myeloma, lymphomas, head and neck squamous cell carcinoma, breast cancer, prostate cancer, and hepatocellular carcinoma (HCC) (23–26). STAT3 generates two isoforms STAT3α and STAT3β by alternative splicing (27). STAT5 consists of two isoforms, STAT5a and STAT5b which map to chromosome17q11.2 in human and share 94% structural homology, these two isoforms are transcribed from separate genes and differ primarily at their C-terminus (19, 28). STAT5a is predominantly expressed in mammary gland and mammary tissue while STAT5b is more prevalent in muscle and liver (19, 28, 29). STAT3, STAT5a, and STAT5b belong to a family of STAT proteins along with STAT1, STAT2, STAT4, and STAT6 (30, 31). STAT proteins were discovered initially as latent transcription factors found in the cytoplasm of cells (32, 33). All seven STAT proteins share a common structural motif (Figure 1).

**Figure 2:**
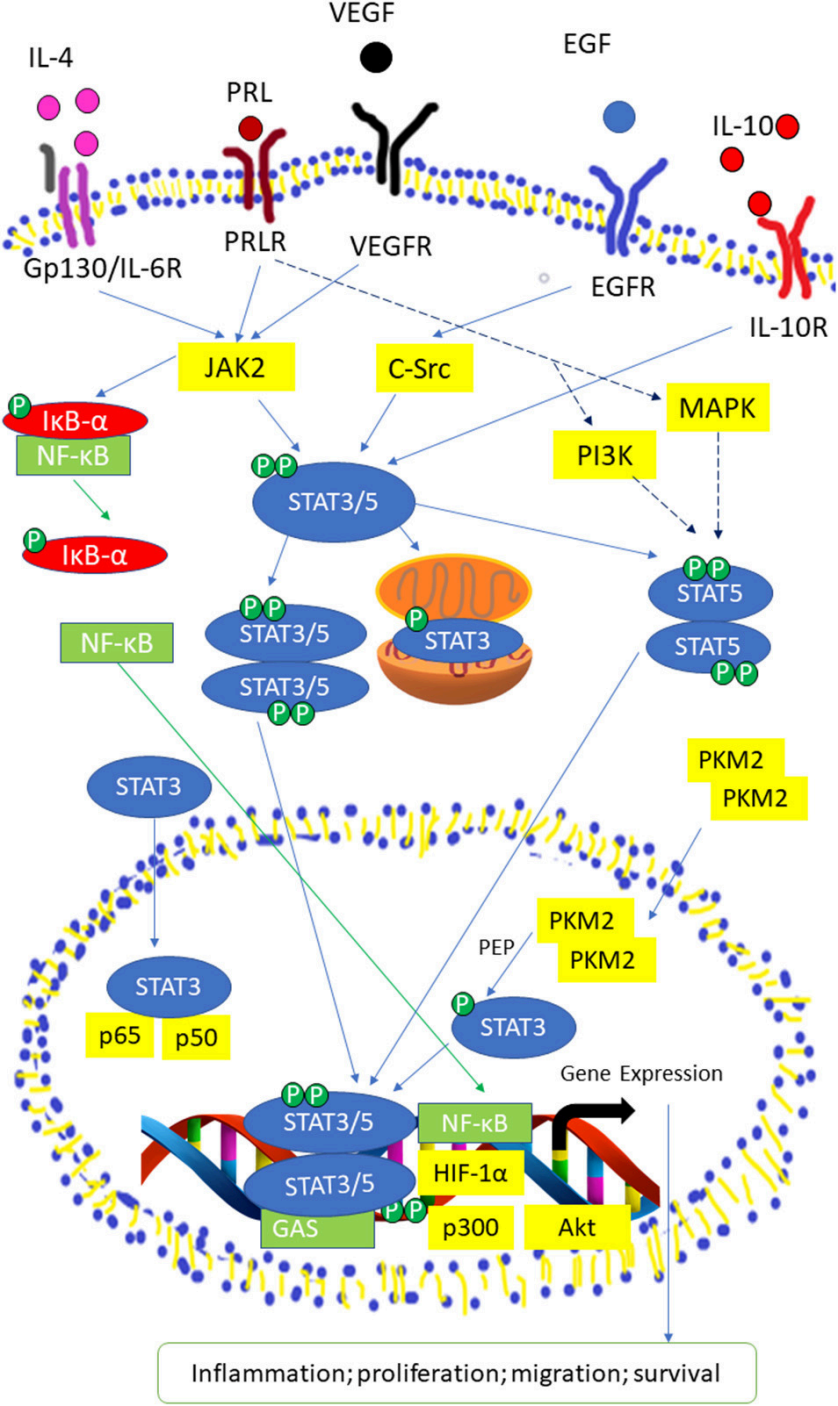
Activated STAT3/5 signaling pathway by IL-6 receptor, Prolactin (PRL) receptor, VEGF Receptor, EGF Receptor, and IL-10 receptor. STAT3 and STAT5 are activated through tyrosine phosphorylation, then the STAT3/5 moomer undergoes dimerization and translocated into the nucleus. In the nucleus, STAT3/5 dimers binds to the promoter region or γ-interferon activation sequence (GAS) of target genes with co-factors, such as Nuclear Factor Kappa (NF-κB), Hypoxia-inducible factor 1-alpha (HIF-1α), protein kinase B (Akt), and p300 to transcript genes related to inflammation, proliferation, migration, and survival. NF-κB is activated through the phosphorylation of Inhibitor of kappa (IκB-α). PRLR activate Mitogen-activated protein kinase (MAPK) and Phosphoinosite 3-kinase (PI3K) pathways to produce co-regulators that are required for expression of certain STAT5-related genes.

STAT3, STAT5a, and STAT5b can be activated by numerous cytokines and growth factors, including interleukin (IL)-6, EGF, insulin-like growth factor, hepatocyte growth factor, colony-stimulating factor-1, platelet derived growth factor, hormones (growth hormone, insulin) and downstream of some G-protein-coupled receptors (34–37). The binding of these molecules will activate JAK kinases and causing the phosphorylation of tyrosine residues on receptors which allow the SH2 domains of STAT proteins to attach to the phosphorylated receptor (38–40). It is crucial for STAT proteins to undergo tyrosine phosphorylation for homodimerization or heterodimerization through an SH2 domain-mediated mechanism (40). Particularly, IL-6 is important for the production of HCC (41, 42) and is able to activate STAT3 via IL-6 receptor, gp130 and JAKs (43, 44). Upon attaching of IL-6 to its receptor, dimerization of the gp130 receptor occurs and causes subsequent activation of JAK due to its association with gp130 (25, 45, 46). Three members of the JAK family of proteins, JAK1, JAK2, and TYK2 can be activated by this manner (47, 48). After which, phosphorylation of tyrosine residues on gp130 is induced, providing docking sites for inactive STAT3 monomers (49, 50) causing STAT3 to be phosphorylated at its Tyr^705^ residue (23, 51). Activated STAT3 monomers then dimerize by coupling of the phosphorylated Tyr^705^ remainder on STAT monomer with Src homology 2 (SH2) domain of another STAT monomer (52, 53). Nuclear translocation happens next, which is mediated by importin α5/NPI-1 (54, 55). Thereafter, STAT3 dimers attach to STAT3-specific DNA-response elements of desired genes and this brings about transcription of genes (56, 57) Phosphorylation of STAT3 can be induced by c-Src directly or indirectly, in which c-Src may exerts its function downstream following activation of G protein-coupled receptor (GPCRs) or receptor tyrosine kinases (RTKs) (47, 58). Apart from its Tyr^705^ residue, STAT3 can be phosphorylated at its Ser^727^ as well. BCR-ABL primarily phosphorylates STAT3 at Ser^727^ while phosphorylation at Tyr705 is at a comparatively lesser degree (58). Additionally, the phosphorylation of the Ser^727^ residue can be moderated by the Ras/mitogen-activated protein kinase (MAPK) pathway when it is induced by IL-6 (36, 59). However, the phosphorylation of Ser^727^ residue on STAT3 is not affecting the DNA-binding ability of STAT3 (60, 61) (Figure 2).

It was reported that STAT5 can be triggered by cytokines, such as Prolactin (PRL), growth hormone, erythropoietin, thrombopoietin, EGF, IL-2, IL-3, IL-6, IL-7, IL-9, and IL-15 (62). STAT5a and STAT5b are phosphorylated at its Tyr^694^ and Tyr^699^ residue by JAK2 (28). STAT3 and STAT5 isoforms are able to be phosphorylated and consequently initialized by non-receptor tyrosine kinases of the Src kinase families (63, 64), particularly c-Src (65, 66). However, activation of STAT5 by Src kinases will only translocate STAT5b into the nucleus Serine phosphorylation sites for STAT5 isoforms are Ser^725^ and Ser^730^ for STAT5a and STAT5b, respectively in murine (67). Another study demonstrated that Ser^779^ in STAT5a was constitutively phosphorylated in multiple tissues (40, 68). Xue et al. (40) demonstrated that both STAT5 isoforms underwent rapid serine phosphorylation in response to IL-2 where the phosphorylation site in STAT5a was Ser^780^ which is not conserved in STAT5b. The mutation of Ser^730^ in STAT5b stimulated the STAT5 DNA binding activity thus promote the growth hormone-induced β-casein promoter activity (69).

## Crosstalk between STATs with Other Transcription Factors in Cancer and Inflammation

Constitutively activated STAT3 and STAT5 are causes of concern due to their tumorigenic potential. Various oncogenic pathways are regulated by STAT3, including cell-cycle progression, apoptosis, angiogenesis, invasion, and metastasis (58, 70). This is because STAT3 is capable of modulating the expression of proteins involved in these pathways, for instance, cell cycle regulator cyclin D1, anti-apoptotic proteins B-cell lymphoma 2 (Bcl-2) and B-cell lymphoma-extra Large (Bcl-xL), Vascular Endothelial Growth Factor (VEGF) (58, 63) and Matrix Metalloproteinase-9 (MMP-9), which performs a main role in invasion (23, 71). Like STAT3, STAT5 is able to modulate growth and suppression of cell death in a few cancer cell lines, notably association with the Bcl-Ab1 fusion protein in hematopoietic cancers (72, 73). STAT5 is reported to influence the expression of Bcl-2, Survivin, MMP-2, MMP-9, VEGF, and E-cadherin by interacting with MAPK pathway in colorectal cancer cells (72). Interestingly, STAT family also collaborate with other transcription factors, such as RUNX family proteins, p53 and Nuclear factor-κB, as described below.

### RUNX Family Proteins

Runt-related (RUNX) family genes were shown to participate in the progression of human cancers and induce tumors in mouse models (74). The RUNX genes exhibit the properties of oncogenes and tumor suppressors in various cancers and cellular contexts (75). All RUNX family genes attach DNA via the conserved Runt domain, and contain the same heterodimeric binding cofactor, CBFβ (75). Mutations in RUNX genes have been correlated with different types of cancers. As one of the developmental regulators in haematopoiesis, functional dysregulation of RUNX1 will lead to leukemia (74, 76). RUNX2 is crucial in breast and prostate cancer progression and is related with osteosarcoma (77, 78). RUNX3 is strongly implicated as tumor suppressor for gastric cancer and the absence of RUNX3 expression is proposed to be linked to gastric cancer because expression of RUNX3 is not observed in more than 45% of the gastric cancer patients (74, 77, 79). JAK-STAT signaling pathway induces interferon-γ (IFN- γ) to express TGF-β signaling pathways inhibitor, Smad7 (80). JAK-STAT signaling pathway indirectly affect the expression of RUNX3 as this gene is closely linked to TGF- β signaling pathways (81–83). By indirect binding of STAT3 and bone morphogenetic proteins (BMP)-specific Smads, BMP pathways interact with Leukemia inhibitory factor (LIF) induce differentiation of astrocytes from neuroepithelial cells (84). Latent form of STAT1 protein binds to RUNX2 and retains it in the cytoplasm thus attenuating its function as a transcription factor in osteoblast and this interaction occurs independent of IFN signaling since phosphorylation of tyrosine-701 of STAT1 is not needed (85). STAT1 is suggested to regulate the role of other RUNX proteins and STAT1 indirectly regulate TGF-β superfamily signaling through the RUNX proteins (86).

### p53

The p53 gene is a tumor suppressor gene and a potent inhibitor of cell growth (87). The p53 protein arrests cell cycle progression at several points and promotes cell death of cancerous cells (88). Activated Stat3 was reported to bind to the p53 promoter both *in vitro* and *in vivo*, supressing the p53 activity in breast cancer cells (88). Both STAT1 and p53 activate the p21 gene promoter (89, 90) and they both bind to p300/CREB binding protein (CBP) at different sites suggest that they may form a complex with CBP to regulate p53-dependent apoptotic signaling pathway (91). Knockout STAT1 gene from a p53-deficient animals leads to rapid tumor development and with a wider spectrum of tumor types (92). Mdm2 protein promotes degradation of the p53 protein by ubiquitination (93), is downregulated by STAT1 (91). Townsend et al. (91) also demonstrated that STAT1 and p53 cooperate in inducing apoptosis in cancer cells. Elevated STAT1 transcription activation is reported to enhance the cytotoxic activity of p53 inducers, such as fludarabine (94). Youlyouz-Marfak et al. (95) showed that agents that are able to induce p53 activation could also promote collateral STAT1 activation. The role of STAT2 in cancer progression and the correlation with p53 remain understudied. Recently Gamero et al. (96) showed that STAT2 is tumorigenic in the absence of p53, STAT2 knockdown in p53 null tumor cells increased protein levels of the marker of epithelial-mesenchymal transition (EMT), E-cadherin while overexpression of STAT2 in p53 null cells reduced E-cadherin protein.

### Nuclear Factor Kappa B

Nuclear factor-κB (NF-κB) is a well-known signaling pathway accountable for inflammation-induced carcinogenesis and anti-tumor immunity. NF-κB has potential of enhancing expression of different inflammatory mediators by acting as their transcription factor in number of immune reactions, it has been designated as major signaling pathway involved in development of tumor as a result of inflammation (97–99). Alongside, dominant part of STAT proteins especially STAT3 in development of inflammation mediated cancers, it is not surprising to mention that it possesses secret crosstalk with NF-κB (100–104). These proteins are constantly expressed in carcinoma cells and indispensable for converting cytoplasmic signals from extracellular stimuli and act as nuclear transcription factors needed for modulating genes involved in tumor growth, existence, angiogenesis and invasiveness, as well as genes encoding key cancer-promoting inflammatory mediators (52, 98, 99, 105, 106). Various inflammatory factors encoded by NF-κB target genes, most notably IL-6, are critical activators of STAT3 (1, 100, 101, 107). While in certain tumors, STAT3 encounters directly with NF-κB to capture it in nucleus and thus causing its activation in malignancies (102). Eventually, STAT3 and NF-κB are major regulators of oncogenic and pro-inflammatory genes (105, 106, 108, 109). In comparison to regulated expression of STAT3 and NF-κB in healthy cells, persistent restricted expression of these genes by uninterrupted activation of STAT3 and NF-κB will results in chronic inflammation and advancement of cancer cell growth (110).

## Therapeutic Implications of Targeting STATs

### JAK-STAT Inhibitor

Recently, Debio 0617B is the first-in-class kinase inhibitor that targeting phospho-STAT3 (pSTAT3) and/or pSTAT5 specifically in carcinomas by suppressing the activity of JAK, SRC, ABL, and class III/V receptor tyrosine kinases (RTK). Debio 0617B demonstrated dose-dependent inhibition of pSTAT3 in STAT3-activated cancer cell lines as well as suppressing cell proliferation in several cancer cell lines and in malignant xenografts derived from patients (111). In year 2011, Ruxolitinib (Jakafi®, Incyte Corp.) was the first United State Food and Drug Administration (USFDA) approved JAK inhibitor which targeting both JAK1 and JAK2 (112). Ruxolitinib was investigated in Clinical Trial Phase 3 as intervention for Myeloproliferative Neoplasms, Polycythemia Vera, Primary Myelofibrosis and Graft-vs-host Disease in year 2018. In comparison with other JAK family members which are ubiquitously expressed in mammals, JAK3 is predominantly expressed in hematopoietic cells and is highly regulated with cell development and activation (113, 114). Signals relayed by the JAK3 protein regulate the growth and maturation of T cells and natural killer cells. Tofacitinib (Xeljanz®, Pfizer) was approved by the USFDA as a JAK3-selective suppressor for rheumatoid arthritis (RA) therapy in year 2012 and recently approved to treat Ulcerative Colitis (112). However, Tofacitinib still demonstrates restricted selectivity against JAK1 and JAK2 which could lead to outcomes like anemia and neutropenia due to simultaneous suppression of JAK1 and JAK2 (115, 116). Pei et al. developed a 4-aminopiperidine-based compound, RB1 which was extremely selective for JAK3, and reasonable pharmacokinetics properties (*F* = 72.52%, T1/2 = 14.6 h) and favorable results of toxicology experiments exhibited by RB1 indicating that it might be a potent candidate for RA treatment (113). Several JAK inhibitors which are currently under developmental phases have been listed in Table 1.

**Table 1:** JAK inhibitors in clinical trial (phase I not included).

**JAK inhibitor**	**Target of the inhibitor**	**Disease in clinical trial**	**Phase of clinical trial**	**ClinicalTrial.gov (USA) number**
Ruxolitinib (INC424)	JAK1,2	Myeloproliferative neoplasms (MPN)	III	NCT00952289
		Polycythemia vera (PV)	III	NCT01243944
		Primary myelofibrosis (MF)	III	NCT02087059
				NCT00934544
			II	NCT01732445
				NCT01795677
				NCT01787552
				NCT01693601
		MF, Post-PV, Post-essential thrombocythemia (ET) myelofibrosis	III	NCT01969838
			II	NCT01392443
				NCT01445769
				NCT00509899
		MF, Acute lymphoblastic leukemia (ALL), MPN, Myelodysplastic syndrome (MDS)	II	NCT02158858
		Graft-vs-host disease (GVHD)	III	NCT03112603
			II	NCT02997280
				NCT03395340
				NCT02953678
		Vitiligo	II	NCT03099304
		ALL	II	NCT02723994
		AML/ALL	II	NCT01251965
		Myelomonocytic leukemia	II	NCT01776723
		T-cell ALL	II	NCT01712659
		Multiple myeloma	II	NCT00639002
		CML	II	NCT01751425
		Hodgkin's lymphoma	II	NCT02164500
				NCT01877005
		Atopic dermatitis	II	NCT02001181
Tofacitinib (CP-690,550)	JAK1,2,3	RA	III	NCT02281552
				NCT00853385
			II	NCT00147498
		Psoriasis	III	NCT01309737
				NCT01815424
				NCT01186744
				NCT01976364
		Arthritis, RA	III	NCT01039688
		Ulcerative colitis	III	NCT01458574
		Alopecia areata (AA), Alopecia totalis (AT), Alopecia universalis (AU)	II	NCT02197455
		Kidney transplantation	II	NCT00658359
				NCT00483756
INCB052793	JAK1	Solid tumors, advanced malignancies, metastatic cancer	II	NCT02265510
AZD4205	JAK1	Non-small cell lung cancer	II	NCT03450330
TD-1473	JAK1,2,3	Crohn's disease	II	NCT03635112
Givinostat (ITF2357)	JAK2	MF	II	NCT00606307
		PV	II	NCT00928707
		MPN	II	NCT01761968
Pacritinib	JAK2	MF	II	NCT03645824
Decernotinib (VX-509)	JAK3	RA	II	NCT01590459
				NCT01754935
Baricitinib	JAK1,2	GVHD	II	NCT02759731
		Giant cell arteritis	II	NCT03026504
Lestauritinib (CEP-701)	JAK 2	MF	II	NCT00494585
BMS-911543	JAK2	MF	II	NCT01236352

Persistent initiation of STAT3 and STAT5 are described in a few human cancer cell lines, and clinical samples (117, 118). However, the development of STAT5 inhibitor has been distinctly slower compared to STAT3, but recent years witness a tremendous effort to fill in this gap (119). For instance, Wingelhofer et al. demonstrated that STAT5 SH2 domain inhibitor, AC-4–130 could directly bind and disturb STAT5 activation, dimerization, nuclear translocation, and STAT5-dependent gene transcription in acute myeloid leukemia (120). On the other hand, a psychotropic drug, pimozide was reported to decrease STAT5 tyrosine phosphorylation, induce cell cycle arrest and cell death in chronic myelogenous leukemia cells (121). In fact, several STAT5 inhibitors in development have advanced to clinical studies and we have summarized these trials in Table 2.

**Table 2:** STAT5 inhibitors in clinical trial.

**STAT5 inhibitor**	**Disease in clinical trial**	**Phase of clinical trial**	**ClinicalTrial.gov (USA) number**
Pioglitazone	CML	II	NCT02888964
Methotrexate and sirolimus	ALL	II	NCT01162551
Tacrolimus	GVHD	II	NCT01927120
Sirolimus			
AT9283	Leukemia	I	NCT01431664

Several natural-derived pharmacological agents have been used to target aberrant JAK/ signaling pathway by diverse mechanism(s) including blockage of upstream tyrosine kinases that can phosphorylate STAT3/5; activation of negative regulators of STAT3/5 signaling cascade; abrogation of STAT3/5 dimerization, acetylation, and DNA binding (26, 122). However, these natural-derived STAT3/5 inhibitors have exhibited limited efficacy in clinical trials so far, and additional studies are required to clearly establish their utility as a monotherapy or in combination regimens with existing drugs for cancer patients. Few important natural-derived STAT3/5 inhibitors have been summarized in Table 3.

**Table 3:** Natural-derived STAT3/5 inhibitors.

**Inhibitors**	**Target of the inhibitor**	**Disease**	**References**
Cryptotanshinone	STAT3	Prostate cancer	(123)
Capsaicin	STAT3	MM	(124)
Curcumin	STAT3	MM	(125)
Cucurbitacin I	STAT3	Osteosarcoma	(126)
Celastrol	STAT3	MM	(60)
Atriprimod	STAT3	MM	(127)
Sulforaphane	STAT5	CML	(128)

### STAT3/5-Targeting RNAi

Inhibition of STAT3 pathway has been studied by several small molecules through either upstream inhibition of cytokine and growth factors, inhibition of STAT3 dimerization, inhibition of STAT3/STAT3 nuclear translocation or inhibition of DNA binding activity (129). Due to cytotoxicity and limitation of specificity, progress of pharmacological inhibitors of STAT3 has been limited. Targeting STAT3 using small molecule inhibitors or other non-specific methods could trigger many undesirable adverse effects because STAT3 is also expressed in normal tissues and involved in many normal cellular processes (64, 130). Thus, these inhibitors need to be delivered by coupled to delivery agents to increase specificity and avoid causing unwanted side effects.

RNA interference (RNAi) is a valuable research tool for analyzing the function of specific genes in cellular and disease processes and for therapeutic application by specific gene knockdown (131–133). RNAi can be mediated either by inserting small interfering RNAs (siRNAs) directly into the cytoplasm of the host cells or by viral vector expressing short hairpin RNAs (shRNAs) which are processed intracellularly into siRNAs by Dicer (134, 135). After the introducing of siRNAs, they will be integrated into RNA-induced silencing complex (RISC), which one strand will be removed and the RISC will be guided by the remaining antisense RNA strand to stop translation of mRNAs bearing complementary sequences (134). The gene knockdown can lasts for 1 week in splitting cells and for a few weeks in non-splitting cells (132).

Konnikova et al. initially transfected human astrocytoma cell line with STAT3 siRNA, which subsequently led to apoptosis induction via inhibition of anti-apoptotic genes (Bcl-XL and Survivin) expression (136). Later they found that similar cell death was observed in human gastric, primary glioblastoma, and breast cancer cells after transfection with STAT3 siRNA (137). On the other hand, Xiong et al. transfected the human colorectal cancer cell lines SW1116 and HT29 with STAT5 siRNA and the results indicated that STAT5 is involved in promoting cancer cell growth, cell cycle progression, invasion and migration by modifying expression of Bcl-2, p16, p21, E-cadherin, VEGF, MMP, and p27 genes in colorectal cancer (72). Another study showed that human glioblastoma-astrocytoma U87 cells transfected with STAT5 targeting siRNA, caused in particular suppression of STAT5 genes and STAT5 mediated DNA-binding activity as well as significant inhibition of cell invasion (138). Overall, STAT3/5 targeting siRNA is proven to promote apoptosis, cell cycle arrest, and suppress cancer cell invasion in various carcinoma cell-line models, including prostate, esophageal, hepatocellular, ovarian, laryngeal, breast, and colorectal cancer (139–146).

The main challenge to develop siRNA as therapeutics is to overcome the siRNA poor deliverability because naked siRNA are susceptible to degradation by extracellular RNases in the bloodstream and could not pass through cell membranes because of their large molecular weight and net negative charge (132, 147). To overcome the challenges, nanoparticles are commonly used for the delivery of the naked siRNA since they offer protection to the siRNA and assist in endocytosis. This review also presents an update on the current strategies used to deliver STAT3/5 targeting siRNA using viral vectors and nanoparticulate delivery systems.

## Other Strategies in Targeting STAT3/5

### Viral Vector

Viruses are natural carriers of genetic information. Previous attempt to transduce B and T lymphocytes using retroviruses had only 27.2% transduction efficiency after a single transduction (148), however the combination of lentiviral vector system with LacZ reporter fusion system is able to deliver STAT3 shRNAs with high level of transduction efficiency with average 85% in three lymphoma cell lines (131). Yang's team (149) transfected human pancreatic cell line, SW1990 with LV-STAT3siRNA third generation self-inactivating lentivirus vector, significant decrease of VEGF and MMP-2, decrease of cell growth, and decrease of invasion ability of the cancer cells were observed after the transfection. In another study, a single injection of lentiviral vectors encoding *Stat3*-targeting shRNA were able to downregulate *Stat3*, Survivin, and MMP-2 at the same time impair tumor cell survival and invasiveness in melanoma models (150). A lentiviral vector was constructed to co-express Interferon (IFN) genes and siRNA targeting STAT3 to suppress the proliferation of B16 melanoma cells with over 95% transduction efficiency (151). Adenoviral vector was used in transfecting human lung adenocarcinoma A549 cells with cDNA of carboxyl-truncated STAT5a variant that inhibit STAT5 isomers-mmediated transcription (152). Due to their immunogenicity and limited transgenic capacity, viral vectors are less commonly utilized in STAT3 RNAi delivery compared to non-viral vectors.

### Nanoparticles

**Figure 3:**
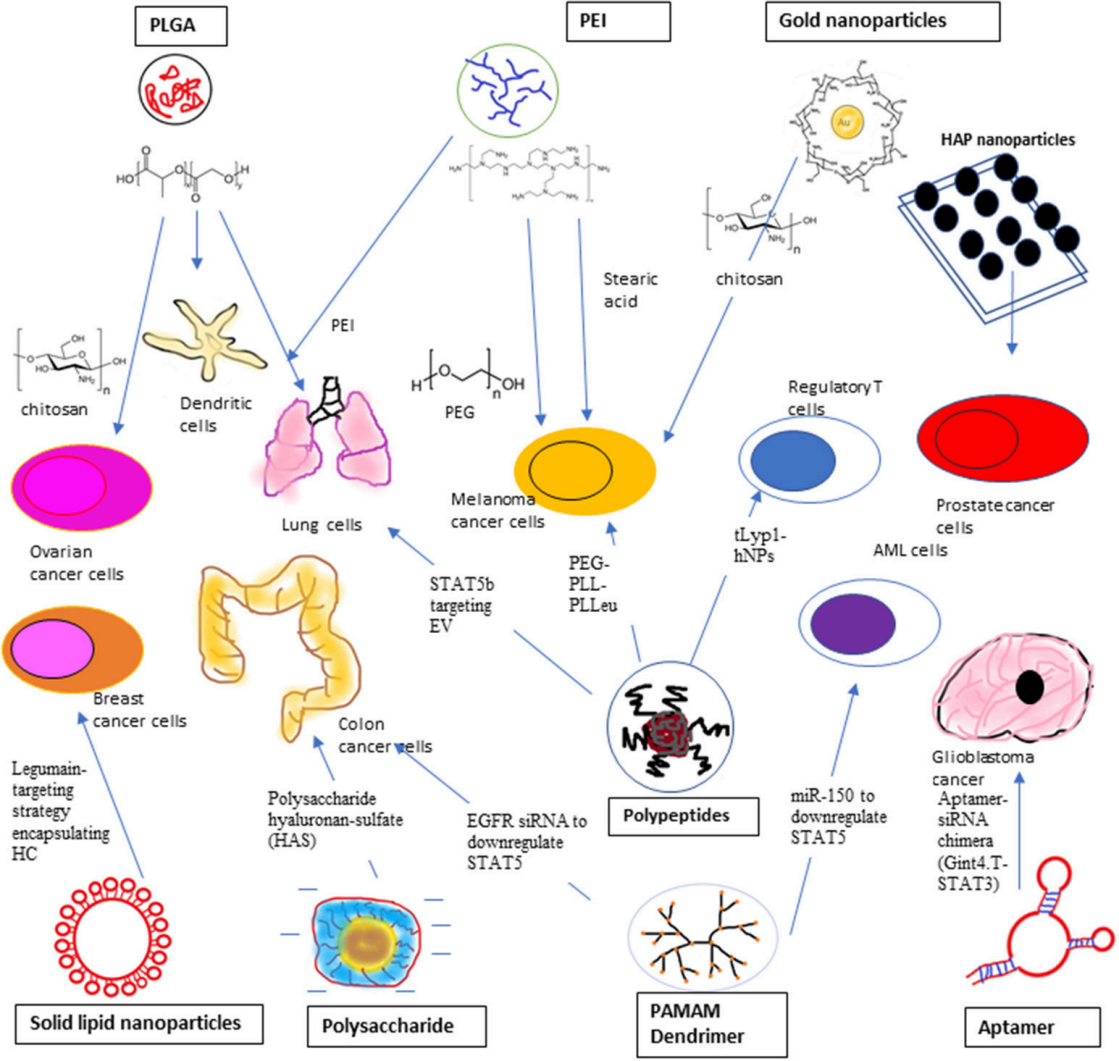
STAT3 siRNA delivery using Polyethyleneimine (PEI), Ploy L-co-glycolic Acid (PLGA), and Inorganic compound, such as gold nanoparticles and Hydroxyapatite (HAP) nanoparticles to target ovarian cancer cells, malfunctioned dendritic cells, lung cancer cells, melanoma cancer cells, and prostate cancer cells. STAT3 siRNA delivery using polypeptides, solid lipid nanoparticles, polysaccharide, and aptamer. Poly (ethylene glycol)-b-poly (L-lysine)-b-poly (L-leucine) (PEG-PPL-PLLeu) is used to target melanoma cells while Triethylamine hybrid nanoparticles (tLypl-hNPs) targets Regulatory T-cells. Legumin-targeting strategy is employed to targeting breast cancer cells using encapsulated hydrocarbon carrier. Anionic charged siRNA co-assembled with polysaccharide hyaluronan-sulfate (HAS) to target mouse colon cancer cells. Gint4.T-STAT3 was developed to deliver STAT3 siRNA to PDGFRβ^+^ Glioblastoma cancer cells. PAMAM Dendrimer was used to deliver EGFR siRNA and miR-150 to suppress STAT5 expression in colon cancer cells and AML cells, respectively. Polypeptide PEGylated-anisamide-LPH nanoparticle was used to transfect lung cancer cells with STAT5B targeting non-apeptide, EV.

Non-viral vectors have the edges of low toxicity, easy to be synthesized and mild immune response over viral vectors to introduce siRNA (153). Non-viral siRNA vectors including liposomes and lipid-like materials, polymers, such as polyethyleneimine (PEI), poly D, L-lactic-co-glycolic acid (PLGA), poly (alkyl cyanoacrylate), chitosan and dendrimers, such as PAMAM, positively charged ionic cell penetrating peptides (CPP), and siRNA bioconjugates, such as cholesterol, antibodies, and aptamers (154). siRNA–PLGA/CSO micelles displayed better cellular uptake and STAT3 gene silencing effectiveness in SKOV3 ovarian cancer cells in comparison with siRNA/CSO complexes at the same N/P ratios with no remarkable differences with lipofectamine 2000 (154). Conventional transfection carriers including polymers and cationic lipids have high cytotoxicity although their transfection efficiency is high. For the cell penetrating peptide based transfection carriers, their efficiency is lower than that of existing lipidic agents due to endosomal trapping although they exhibit better cytotoxicity profiles (155). By incorporating high molecular weight polyethyleneimine cationic lipids into membrane bilayers within the cells can stimulate the introduction of siRNA into the cytoplasm, but it is also generating reactive oxygen species (ROS) and Ca^2+^ discharge (156). Usage of cationic lipids or polymers raised concerns about their suitability for systemic delivery as the result of serum instability, carrier aggregation, and cytotoxicity (157–159). This also lead to investigation of the feasibility of anionic nanoparticles for siRNA delivery (158). We have summarized the STAT3/5-Targeting Nanoparticles in Figure 3.

#### Polyethyleneimine (PEI)

Due to its high toxicity, PEI is not preferable to be utilized as non-viral vector for siRNA delivery (154). To overcome the high toxicity and better STAT3 silencing effect, PEI was modified with stearic acid (StA) to deliver STAT3 siRNA to induce tumor apoptosis in B16 melanoma cells, it showed higher potency in STAT3 silencing with a significant induction of IL-6 secretion, 2.5 times higher expression of cellular Caspase3 and reduction of VEGF production, in comparison to PEI complexes alone (129). The modification of branched PEI with lipids will protect siRNA integrity and improve the siRNA delivery into the cytoplasm (160). The complex of siRNA/PEI/StA showed significant increase of IL-6 secretion, increase of cellular Caspase3 activity and reduction of VEGF production compared to siRNA/PEI (129). PEI also was used in combination with PLGA (161, 162) and Graphene oxide (163). Graphene oxide was functionalized with PEI and polyethylene glycol (PEG) to serve as a plasmid based *Stat3* siRNA carrier in the *in vivo* study of treating mouse melanoma cell line (163).

#### Poly L-lactic-co-glycolic Acid (PLGA)

PLGA has been widely used as non-viral carrier in STAT3 siRNA delivery in different combinations in recent year. Positively charged Chitosan oligosaccharide (CSO) was added to the STAT3 siRNA-PLGA micelles and the micelles showed high efficiencies of cellular uptake and STAT3 gene silencing in SKOV3 ovarian cancer cells (154). Chitosan was chosen as a condensing agent due to its unique biological activities and when it is fused with siRNA-PLGA conjugates, it can form smaller particles to increase the cellular uptake (154). PLGA nanoparticles can be directly coated with cationic polymers, such as PEI which allow siRNA to attach on their surfaces (164). Combination of non-toxic PLGA and PEI nanoparticles could deliver siRNA cross the blood brain barrier, promoted cell death and arrested cells at G1/G0 stage *in vitro* and *in vivo*, lead to notable reduce in expression of IL-6 and the angiogenic factor and elevate in caspase3 activity in tumor bearing mice (161). Su's team developed a novel strategy to synthesize PEI-coated paclitaxel-loaded PLGA nanoparticles to target human lung malignant cells and paclitaxel-resistant cell lines (162). *Stat3*-enhanced chemoresistance in lung malignant cells were suppressed by introduction of *Stat3* siRNA thus causing the tumor cells more responsive to paclitaxel (162).

PLGA nanoparticles that containing both STAT3 siRNA, an immune response modifier (imiquimod, R837) and near-infrared (NIR) fluorophores (indocyanine green) enable researchers to monitor the migration of the activated Dendritic cells after the transfection using real time NIR fluorescence imaging (165). Other than siRNA, STAT3 inhibitor JSI-124 was chemically conjugate to PLGA, generated a conjugate which exhibited potent anticancer and STAT3 silencing in immunosuppressed dendritic cells (166). The PLGA-JSI-124 conjugate worked best when combined with Dendritic cells adjuvant CpG (166).

#### Inorganic Compounds

To enhance the stability and cell uptake, layer-by-layer chitosan coated gold nanoparticles were used to deliver STAT3 siRNA and co-deliver *Stat3* siRNA and Imatinib (IM) to treat murine melanoma cells using iontophoresis to strengthen the localized skin penetration (167, 168). Gold nanoparticles have demonstrated potential in biomedical application because they have high biocompatibility, chemically inert, nanosized, versatile and have longer plasma circulation (169). In the treatment of melanoma cells, it was the first report to demonstrate *in vivo* efficacy studies of non-invasive iontophoretic administration of anti-cancer agents that was comparable with intratumoral administration (151). Layer-by layer assembly of polyelectrolytes formed a firm entrapment of siRNA and IM at the same time the electrostatic interactions facilitated adequate slow deliver of therapeutics (151). Plasmid-based *Stat3* siRNA introduced by CaCl_2_ modified Hydroxyapatite (HAP) nanoparticles was able to lower the protein expression of *Stat3* and *p-Stat3* in prostate tumor bearing mice at the same time downregulated the expression of *Stat3*-associated downstream genes (170). A hybrid of lipid and polymer vesicles with calcium phosphate as the solid kernel (CaP@HA) was used to introduce STAT3-specific decoy oligonucleotides (STAT3-decoy-ODNs) into TRAZ-resistant HER2-positive breast cancer cells (171). ODNs packaged with CaP@HA showed significantly increased serum stability, cellular transfection, synergistic cytotoxicity and apoptosis *in vitro* compared to normal ODNs (171).

#### Polypeptides

Poly (ethylene glycol)-b-poly (L-lysine)-b-poly (L-leucine) (PEG-PLL-PLLeu) polypeptide micelles was used for co-encapsulating Toll-like receptor agonist, STAT3 siRNA and OVA antigen to generate nanovaccine in OVA-transfected melanoma cell line (172). PMP/OVA/siRNA simultaneously facilitated the cellular uptake of OVA antigen and siRNA about 3–200-folds, and decreased STAT3 expression in TADCs over 50% both *in vitro* and in *vivo* (172). Melittin derived peptides, P5RHH could conjugated with *Stat3* siRNA to form nanoparticles with small size (190 nm in diameter) with negligible cytotoxicity (155). p5RHH/STAT3 siRNA nanoparticles mediated transfection to impede malignant cell growth, angiogenesis and foam cell formation in mouse melanoma cells while maintaining their size and transfection effectiveness even there are serum proteins around (155).

tLyp1 peptide-conjugated with PEG-DSPE and triethylamine to form hybrid nanoparticles (tLyp1-hNPs) for targeting Treg cells by suppressing of STAT3 and STAT5 phosphorylation at the same time improve the effect of imatinib (173). Imatinib (IMT) has been shown to downregulating Treg cell expression but its application was limited by poor solubility and high cytotoxicity (174). Treg cell targeted tLyp1-hNPs also showed improved survival rate, improved tumor suppression, reduced intratumoral Treg cells, and increased intratumoral CD8^+^ T cells against malignant cells in *in vivo* study when incorporate with anti-cytotoxic T-lymphocyte antigen-4 (CTLA4) antibody (173).

King and Huang (175) developed EEEEpYFELV (EV), a non-apeptide that inhibit STAT5b phosphorylation and they delivered the inhibitor using a PEGylated-anisamide-LPH nanoparticles to target human lung cancer cells. EV also demonstrated inhibition effect on the phosphorylation of STAT5a without affecting STAT3 phosphorylation (175).

#### Solid Lipid Nanoparticles

Curcumin can exert anti-inflammatory effects in colitis by inhibiting NK-κb activation and STAT3 pathway (176). A synthetic analog of curcumin, Hydrazinocurcumin (HC) was synthesized to improve its stability, water solubility, cell permeability and bioavailability to surpass curcumin (177). HC were mixed with 1,2-Dioleoyl-sn-glycero-3-phosphoethanolamine (DOPE), Dipalmitoylphosphatidylcholine (DOPC), Cholesterol and DOPE-PEG to generate peptide-lipid conjugated HC nanoparticles to treat breast cancer cell line (178). The study showed that HC encapsulated nanoparticles effectively convert tumor associated M2 macrophage to M1 macrophage with reduced expression of IL-10, TGF-β, p-STAT3, MMP-9, MMP-2, and VEGF, along with elevated expression of IL-12 (pro-inflammatory cytokines) in 4T1 murine breast cancer cell line (178). To enhance targeting capability and solid-tumor penetration, Legumain-targeting strategy encapsulating HC were used by adding Legumain-targeted RR-11a into the liposomal nanoparticles (178, 179). In another study, the suitability of introducing Curcumin in solid lipid nanoparticles (SLN-curc) or d-α-Tocopheryl polyethylene glycol 1,000 succinate (TPGS) nanoparticles (TPGS-curc) was investigated in Hodgkin's lymphoma in mice, which SLN-curc was more superior compared to TPGS-curc in term of pharmacokinetic profile (180).

Non-toxic cationic solid lipid nanoparticles (SLN) was combined with STAT3 decoy oligodeoxynucleotides (ODN) to treat human ovarian cancer cells and cellular uptake of SLN-decoy ODN was comparable to that of Lipo-decoy ODN which is more toxic (181). SLN-STAT3 decoy ODN complexes increased expression of cleaved caspase 3, Bax, Beclin-1 and LC3-II and reduced expression of Bcl-2, pro-caspase 3, Survivin, p-Akt, and pmTOR in human ovarian cancer cell lines (181). Overexpression of STAT3 in Non-Small Cell Lung Cancer (NSCLC) patients is a strong predictor of poor prognosis, cationic solid lipid nanoparticles (cSLN) were used to deliver RNAi-mediating plasmid DNA targeting STAT3 in cisplatin resistant lung cancer cells (182, 183). STAT3 mRNA expression level were lowered by ~5-fold in chemo-resistant Calu1 NSCLC cells after treatment of cSLN:plasmid DNA complexes (K2 and K3) (182).

Reconstituted HDL (rHDL) nanoparticles were utilized to deliver STAT3 siRNA or FAK siRNA to treat human ovarian and colorectal cancer cell lines (184). Combination treatment with STAT3 siRNA/rHDL and docetaxel further impede malignant cell proliferation: Treated the cells with Docetaxel independently caused no impact on cell proliferation, meanwhile STAT3 siRNA/rHDL monotherapy resulted in 19% reduction of proliferation (184).

#### Polysaccharides

Although anionic charged nanoparticles were less commonly used in siRNA delivery, one research group introduced anionic siRNA nanoparticles co-assembled with polysaccharide hyaluronan-sulfate (HAS) mediated by calcium ion bridges (158). Effective cellular uptake of this anionic siRNA nanoparticles, together with strong gene silencing was observed in several cell types, which include murine primary peritoneal macrophages and human hepatocellular malignant cells (158). Anionic complexes generated via reversible complexation of siRNA and calcium ions in solution were able to downregulate various genes in several cell lines (185). The semi-synthetic HAS was selected because it has multiple functional groups available for ligand attachment and it is more stable compared to non-modified hyaluronan (186). Alginate sulfate (AlgS) with STAT3 siRNA were co-assembled and then followed by bioconjugation of N-acetylgalactosamine (GalNAc) which is specific to the asialoglycoprotein receptors that are overly expressed on hepatocytes to produce a novel tumor specific delivery vector (GalNAc-NPs) (187). GalNAc-NPs enter the cells through ASGPR-mediated endocytosis, these NPs able to induce 67% reduction in STAT3 mRNA levels at 2.5 mM Ca^2+^ (187).

#### Aptamers

Aptamers can be introduced into the host cells specifically through cell-mediated endocytosis, solving probable problems of other oligonucleotide therapeutics, such as siRNA, shRNA and miRNA (188). A novel aptamer-siRNA chimera (Gint4.T-STAT3) was developed to deliver STAT3 siRNA efficiently and subsequently suppressed the expression of STAT3 in PDGFRβ^+^ glioblastoma multiforme (GBM) cells, through *in vitro* and *in vivo* studies, demonstrating that this is an effective strategy (189). Gint4.T-STAT3 possesses good serum stability and reduces the expression of STAT3 and its target genes (cMYC, MCL-1, Bcl-2, and Bcl-XL). Moreover, a reduction of pro-caspase 3 and Bcl-XL anti-apoptotic protein levels was also observed (189). PDGFRβ is a receptor tyrosine kinase (RTK) which is frequently found in GBM (190). Esposito et al. showed that Gint4.T is capable of passing through a tri-culture *in vitro* model of hematoencephalic barrier, thus making it a suitable carrier targeting GBM cancer stem-like cells (189).

#### PAMAM Dendrimer

Polyamidoamine (PAMAM) dendrimers are one of the most popular dendrimers. In one study, EGFR antisense oligonucleotides were encapsulated with PAMAM dendrimers to targeting colon cancer cell line (191). The expression level of EGFR was significantly reduced up to 40–50%, consequently the downstream genes of EGFR pathway, MAPK1, and STAT5 expression were down-regulated (191). Jiang et al. (192) developed a nanoparticle delivering system by conjugating FLT3 ligand, amidoamine and PAMAM and used this nanoparticle to deliver miR-150 into AML cells. The inhibition of FLT3 signaling pathway by this nanoparticle suppress the activity of ERK, AKT and STAT5 *in vivo* indirectly (192).

#### Exosomes

Exosomes are extracellular membrane vesicles with a diameter of 30–120 nm and containing endogenous proteins, genetic material, such as DNA, and lipids (193). Recently, Zhang et al. (194) demonstrated that two exosome subpopulations, Exo-L and Exo-S contained proteins that are involved in regulating secretion pathways including IL-2/STAT5 signaling pathways, suggesting that these nanoparticles have the potential role for STAT5 targeting treatment.

## Concluding Remarks

In addition to the well-distinguished role of STAT family as transcription factors in causing inflammation and tumorigenesis, we have described various strategies used to regulate STAT3/5 transcription activity either through JAK-STAT inhibitor or RNAi-based targeting system. The findings described in this review indicate that the current understanding of STAT pathway has advanced to the point where different kind of therapeutic interventions are being developed to regulate aberrant activation of STAT protein in order to control chronic inflammation or improve prognosis of several cancers. There is still a need to understand how STAT proteins crosstalk with other signaling pathways and further improve the drug delivery system if we are to develop rational strategies tailored to individual cancers. Based on the progression reported in several studies, STAT3 targeting siRNA using nanoparticulate delivery systems currently offer higher specificity with lesser side effect compared to conventional drug delivery system. These proof-of-concept studies might hold the potential to become mainstream strategies in regulating STAT activation and more works are needed in order to advance these methods into clinical phase.

## Author Contributions

C-YL and CL conceived the review. C-YL, AA, AN, WW, GS, and CL searched the literature, drafted the manuscript, and critically revised it. C-YL and CL designed and coordinated the manuscript. All the authors read and approved the submitted version of the manuscript.

### Conflict of Interest Statement

The authors declare that the research was conducted in the absence of any commercial or financial relationships that could be construed as a potential conflict of interest.

## References

[1] Catlett-Falcone R Landowski TH Oshiro MM Turkson J Levitzki A Savino R. Constitutive activation of Stat3 signaling confers resistance to apoptosis in human U266 myeloma cells. Immunity (1999) 10:105–15. 10.1016/S1074-7613(00)80011-4 10023775

[2] Grandis JR Drenning SD Chakraborty A Zhou MY Zeng Q Pitt AS Requirement of Stat3 but not Stat1 activation for epidermal growth factor receptor- mediated cell growth *in vitro*. J Clin Invest. (1998) 102:1385–92. 10.1172/JCI3785 9769331 PMC508986

[3] Lou W Ni Z Dyer K Tweardy DJ Gao AC. Interleukin-6 induces prostate cancer cell growth accompanied by activation of stat3 signaling pathway. Prostate (2000) 42:239–42. 10.1002/(SICI)1097-0045(20000215)42:3<239::AID-PROS10>3.0.CO;2-G 10639195

[4] Zhang YW Wang LM Jove R Vande Woude GF. Requirement of Stat3 signaling for HGF/SF-Met mediated tumorigenesis. Oncogene (2002) 21:217–26. 10.1038/sj.onc.1205004 11803465

[5] Kaido T Oe H Imamura M. Interleukin-6 augments hepatocyte growth factor-induced liver regeneration; involvement of STAT3 activation. Hepatogastroenterology (2004) 51:1667–70. 15532800

[6] Hung W Elliott B. Co-operative effect of c-Src tyrosine kinase and Stat3 in activation of hepatocyte growth factor expression in mammary carcinoma cells. J Biol Chem. (2001) 276:12395–403. 10.1074/jbc.M010715200 11278729

[7] Garcia R Bowman TL Niu G Yu H Minton S Muro-Cacho CA. Constitutive activation of Stat3 by the Src and JAK tyrosine kinases participates in growth regulation of human breast carcinoma cells. Oncogene (2001) 20:2499–513. 10.1038/sj.onc.1204349 11420660

[8] Bowman T Garcia R Turkson J Jove R. STATs in oncogenesis. Oncogene (2000) 19:2474–88. 10.1038/sj.onc.1203527 10851046

[9] Lin TS Mahajan S Frank DA. STAT signaling in the pathogenesis and treatment of leukemias. Oncogene (2000) 19:2496–504. 10.1038/sj.onc.1203486 10851048

[10] Schwaller J Parganas E Wang D Cain D Aster JC Williams IR. Stat5 is essential for the myelo- and lymphoproliferative disease induced by TEL/JAK2. Mol Cell (2000) 6:693–704. 10.1016/S1097-2765(00)00067-8 11030348

[11] Bromberg JF Horvath CM Wen Z Schreiber RD Darnell JE Jr. Transcriptionally active Stat1 is required for the antiproliferative effects of both interferon alpha and interferon gamma. Proc Natl Acad Sci USA. (1996) 93:7673–8. 10.1073/pnas.93.15.7673 8755534 PMC38805

[12] Shankaran V Ikeda H Bruce AT White JM Swanson PE Old LJ. IFNgamma and lymphocytes prevent primary tumour development and shape tumour immunogenicity. Nature (2001) 410:1107–11. 10.1038/35074122 11323675

[13] Gao SP Mark KG Leslie K Pao W Motoi N Gerald WL. Mutations in the EGFR kinase domain mediate STAT3 activation via IL-6 production in human lung adenocarcinomas. J Clin Invest. (2007) 117:3846–56. 10.1172/JCI31871 18060032 PMC2096430

[14] Ernst M Najdovska M Grail D Lundgren-May T Buchert M Tye H. STAT3 and STAT1 mediate IL-11–dependent and inflammation-associated gastric tumorigenesis in gp130 receptor mutant mice. J Clin Invest. (2008) 118:1727–38. 10.1172/JCI34944 18431520 PMC2323192

[15] Arredondo J Chernyavsky AI Jolkovsky DL Pinkerton KE Grando SA. Receptor-mediated tobacco toxicity: cooperation of the Ras/Raf-1/MEK1/ERK and JAK-2/STAT-3 pathways downstream of α7 nicotinic receptor in oral keratinocytes. FASEB J. (2006) 20:2093–101. 10.1096/fj.06-6191com 17012261

[16] Sano S Chan KS Kira M Kataoka K Takagi S Tarutani M. Signal transducer and activator of transcription 3 is a key regulator of keratinocyte survival and proliferation following UV irradiation. Cancer Res. (2005) 65:5720–9. 10.1158/0008-5472.CAN-04-4359 15994947

[17] O'shea JJ Plenge R. JAK and STAT signaling molecules in immunoregulation and immune-mediated disease. Immunity (2012) 36:542–50. 10.1016/j.immuni.2012.03.014 22520847 PMC3499974

[18] Rauch I Müller M Decker T. The regulation of inflammation by interferons and their STATs. JAKSTAT (2013) 2:e23820. 10.4161/jkst.23820 24058799 PMC3670275

[19] Rani A Murphy JJ. STAT5 in cancer and immunity. J Interferon Cytokine Res. (2016) 36:226–37. 10.1089/jir.2015.0054 26716518

[20] Glosson NL Bruns HA Kaplan MH. Wheezing and itching: the requirement for STAT proteins in allergic inflammation. JAKSTAT (2012) 1:3–15. 10.4161/jkst.19086 24058746 PMC3670132

[21] O'shea JJ Lahesmaa R Vahedi G Laurence A Kanno Y. Genomic views of STAT function in CD4^+^ T helper cell differentiation. Nat Rev Immunol. (2011) 11:239. 10.1038/nri2958 21436836 PMC3070307

[22] Stritesky GL Kaplan MH. Changing the STATus quo in T helper cells. Transcription (2011) 2:179–82. 10.4161/trns.2.4.16614 21922060 PMC3173685

[23] Aggarwal BB Sethi G Ahn KS Sandur SK Pandey MK Kunnumakkara AB. Targeting signal-transducer-and-activator-of-transcription-3 for prevention and therapy of cancer: modern target but ancient solution. Ann N Y Acad Sci. (2006) 1091:151–69. 10.1196/annals.1378.063 17341611

[24] Chai EZP Shanmugam MK Arfuso F Dharmarajan A Wang C Kumar AP. Targeting transcription factor STAT3 for cancer prevention and therapy. Pharmacol Ther. (2016) 162:86–97. 10.1016/j.pharmthera.2015.10.004 26478441

[25] Subramaniam A Shanmugam MK Perumal E Li F Nachiyappan A Kumar AP Possible involvement of signal transducer and activator of transcription-3 (STAT3) signaling pathway in the initiation and progression of hepatocellular carcinoma. In: Perspectives in Cancer Prevention-Translational Cancer Research. New Delhi: Springer (2014). p. 73–87.

[26] Wong ALA Hirpara JL Pervaiz S Eu JQ Sethi G Goh BC. Do STAT3 inhibitors have potential in the future for cancer therapy? Expert Opin Investig Drugs (2017) 26:883–7. 10.1080/13543784.2017.1351941 28714740

[27] Wingelhofer B Neubauer HA Valent P Han X Constantinescu SN Gunning PT. Implications of STAT3 and STAT5 signaling on gene regulation and chromatin remodeling in hematopoietic cancer. Leukemia (2018) 32:1713–26. 10.1038/s41375-018-0117-x 29728695 PMC6087715

[28] Able A Burrell J Stephens JJB. STAT5-interacting proteins: a synopsis of proteins that regulate STAT5 activity. Biology (Basel) (2017) 6:20. 10.3390/biology6010020 28287479 PMC5372013

[29] Hennighausen L Robinson GW. Interpretation of cytokine signaling through the transcription factors STAT5A and STAT5B. Genes Dev. (2008) 22:711–21. 10.1101/gad.1643908 18347089 PMC2394721

[30] Ihle JN. The Stat family in cytokine signaling. Curr Opin Cell Biol. (2001) 13:211–7. 10.1016/S0955-0674(00)00199-X 11248555

[31] Lee JH Kim C Baek SH Ko J-H Lee SG Yang WM. Capsazepine inhibits JAK/STAT3 signaling, tumor growth, and cell survival in prostate cancer. Oncotarget (2017) 8:17700. 10.18632/oncotarget.10775 27458171 PMC5392279

[32] Darnell JE. STATs and gene regulation. Science (1997) 277:1630–5. 10.1126/science.277.5332.1630 9287210

[33] Zhang J Ahn KS Kim C Shanmugam MK Siveen KS Arfuso F. Nimbolide-induced oxidative stress abrogates STAT3 signaling cascade and inhibits tumor growth in transgenic adenocarcinoma of mouse prostate model. Antioxid Redox Signal. (2016) 24:575–89. 10.1089/ars.2015.6418 26649526

[34] Kim C Lee S-G Yang WM Arfuso F Um J-Y Kumar AP. Formononetin-induced oxidative stress abrogates the activation of STAT3/5 signaling axis and suppresses the tumor growth in multiple myeloma preclinical model. Cancer Lett. (2018) 431:123–41. 10.1016/j.canlet.2018.05.038 29857127

[35] Lee JH Kim C Sethi G Ahn KS. Brassinin inhibits STAT3 signaling pathway through modulation of PIAS-3 and SOCS-3 expression and sensitizes human lung cancer xenograft in nude mice to paclitaxel. Oncotarget (2015) 6:6386. 10.18632/oncotarget.3443 25788267 PMC4467444

[36] Rajendran P Li F Shanmugam MK Kannaiyan R Goh JN Wong KF. Celastrol suppresses growth and induces apoptosis of human hepatocellular carcinoma through the modulation of STAT3/JAK2 signaling cascade *in vitro* and *in vivo*. Cancer Prev Res. (2012) 5:631–43. 10.1158/1940-6207.CAPR-11-0420 22369852

[37] Tormo AJ Letellier M-C Sharma M Elson G Crabé S Gauchat J-F. IL-6 activates STAT5 in T cells. Cytokine (2012) 60:575–82. 10.1016/j.cyto.2012.07.002 22854263

[38] Leonard WJ O'Shea JJ. Jaks and STATs: biological implications. Annu Rev Immunol. (1998) 16:293–322. 9597132 10.1146/annurev.immunol.16.1.293

[39] Stark GR Kerr IM Williams BR Silverman RH Schreiber RD How Cells Respond to Interferons. Palo Alto, CA: Annual Reviews (1998). 10.1146/annurev.biochem.67.1.227 9759489

[40] Xue HH Fink Jr DW Zhang X Qin J Turck CW Leonard WJ. Serine phosphorylation of Stat5 proteins in lymphocytes stimulated with IL-2. Int Immunol. (2002) 14:1263–71. 10.1093/intimm/dxf101 12407017

[41] Scheel C Eaton EN Li SH-J Chaffer CL Reinhardt F Kah K-J. Paracrine and autocrine signals induce and maintain mesenchymal and stem cell states in the breast. Cell (2011) 145:926–40. 10.1016/j.cell.2011.04.029 21663795 PMC3930331

[42] Zhang J Sikka S Siveen KS Lee JH Um J-Y Kumar AP. Cardamonin represses proliferation, invasion, and causes apoptosis through the modulation of signal transducer and activator of transcription 3 pathway in prostate cancer. Apoptosis (2017) 22:158–68. 10.1007/s10495-016-1313-7 27900636

[43] Liu Y Fuchs J Li C Lin J. IL-6, a risk factor for hepatocellular carcinoma: FLLL32 inhibits IL-6-induced STAT3 phosphorylation in human hepatocellular cancer cells. Cell Cycle (2010) 9:3423–7. 10.4161/cc.9.17.12946 20818158

[44] Mohan CD Bharathkumar H Bulusu KC Pandey V Rangappa S Fuchs JE. Development of a novel azaspirane that targets the Janus kinase-signal transducer and activator of transcription (STAT) pathway in hepatocellular carcinoma *in vitro* and *in vivo*. J Biol Chem. (2014) 289:34296–307. 10.1074/jbc.M114.601104 25320076 PMC4256360

[45] Baek SH Ko J-H Lee H Jung J Kong M Lee J-w. Resveratrol inhibits STAT3 signaling pathway through the induction of SOCS-1: role in apoptosis induction and radiosensitization in head and neck tumor cells. Phytomedicine (2016) 23:566–77. 10.1016/j.phymed.2016.02.011 27064016

[46] Subramaniam A Shanmugam MK Perumal E Li F Nachiyappan A Dai X. Potential role of signal transducer and activator of transcription (STAT) 3 signaling pathway in inflammation, survival, proliferation and invasion of hepatocellular carcinoma. Biochim Biophys Acta (2013) 1835:46–60. 10.1016/j.bbcan.2012.10.002 23103770

[47] Kim C Cho SK Kapoor S Kumar A Vali S Abbasi T. β-caryophyllene oxide inhibits constitutive and inducible STAT3 signaling pathway through induction of the SHP-1 protein tyrosine phosphatase. Mol Carcinog. (2014) 53:793–806. 10.1002/mc.22035 23765383

[48] Leaman DW Leung S Li X Stark GR. Regulation of STAT-dependent pathways by growth factors and cytokines. FASEB J. (1996) 10:1578–88. 10.1096/fasebj.10.14.9002549 9002549

[49] Hirano T Ishihara K Hibi M. Roles of STAT3 in mediating the cell growth, differentiation and survival signals relayed through the IL-6 family of cytokine receptors. Oncogene (2000) 19:2548. 10.1038/sj.onc.1203551 10851053

[50] Kim SM Kim C Bae H Lee JH Baek SH Nam D. 6-Shogaol exerts anti-proliferative and pro-apoptotic effects through the modulation of STAT3 and MAPKs signaling pathways. Mol Carcinog. (2015) 54:1132–46. 10.1002/mc.22184 24962868

[51] Baek SH Lee JH Kim C Ko J-H Ryu S-H Lee S-G. Ginkgolic acid C 17: 1, derived from Ginkgo biloba leaves, suppresses constitutive and inducible STAT3 activation through induction of PTEN and SHP-1 tyrosine phosphatase. Molecules (2017) 22:276. 10.3390/molecules22020276 28208828 PMC6155672

[52] Haura EB Turkson J Jove R. Mechanisms of disease: insights into the emerging role of signal transducers and activators of transcription in cancer. Nat Rev Clin Oncol. (2005) 2:315. 10.1038/ncponc0195 16264989

[53] Shanmugam MK Rajendran P Li F Kim C Sikka S Siveen KS. Abrogation of STAT3 signaling cascade by zerumbone inhibits proliferation and induces apoptosis in renal cell carcinoma xenograft mouse model. Mol Carcinog. (2015) 54:971–85. 10.1002/mc.22166 24797723

[54] Ma J Cao X. Regulation of Stat3 nuclear import by importin α5 and importin α7 via two different functional sequence elements. Cell Signal. (2006) 18:1117–26. 10.1016/j.cellsig.2005.06.016 16298512

[55] Sethi G Chatterjee S Rajendran P Li F Shanmugam MK Wong KF. Inhibition of STAT3 dimerization and acetylation by garcinol suppresses the growth of human hepatocellular carcinoma *in vitro* and *in vivo*. Mol Cancer (2014) 13:66. 10.1186/1476-4598-13-66 24655440 PMC3998115

[56] Jing N Tweardy DJ. Targeting Stat3 in cancer therapy. Anticancer Drugs (2005) 16:601–7. 10.1097/00001813-200507000-00002 15930886

[57] Lee JH Chiang SY Nam D Chung W-S Lee J Na Y-S. Capillarisin inhibits constitutive and inducible STAT3 activation through induction of SHP-1 and SHP-2 tyrosine phosphatases. Cancer Lett. (2014) 345:140–8. 10.1016/j.canlet.2013.12.008 24333736

[58] Siveen KS Sikka S Surana R Dai X Zhang J Kumar AP. Targeting the STAT3 signaling pathway in cancer: role of synthetic and natural inhibitors. Biochim Biophys Acta (2014) 1845:136–54. 10.1016/j.bbcan.2013.12.005 24388873

[59] Li F Fernandez PP Rajendran P Hui KM Sethi G. Diosgenin, a steroidal saponin, inhibits STAT3 signaling pathway leading to suppression of proliferation and chemosensitization of human hepatocellular carcinoma cells. Cancer Lett. (2010) 292:197–207. 10.1016/j.canlet.2009.12.003 20053498

[60] Kannaiyan R Hay HS Rajendran P Li F Shanmugam MK Vali S. Celastrol inhibits proliferation and induces chemosensitization through down-regulation of NF-κB and STAT3 regulated gene products in multiple myeloma cells. Br J Pharmacol. (2011) 164:1506–21. 10.1111/j.1476-5381.2011.01449.x 21506956 PMC3221104

[61] Wen Z Darnell Jr JE. Mapping of Stat3 serine phosphorylation to a single residue (727) and evidence that serine phosphorylation has no influence on DNA binding of Stat1 and Stat3. Nucleic Acids Res. (1997) 25:2062–7. 10.1093/nar/25.11.2062 9153303 PMC146718

[62] Kazansky AV Kabotyanski EB Wyszomierski SL Mancini MA Rosen JM. Differential effects of prolactin andsrc/abl kinases on the nuclear translocation of STAT5B and STAT5A. J Biol Chem. (1999) 274:22484–92. 10428824 10.1074/jbc.274.32.22484

[63] Rajendran P Li F Manu KA Shanmugam MK Loo SY Kumar AP. γ-Tocotrienol is a novel inhibitor of constitutive and inducible STAT3 signalling pathway in human hepatocellular carcinoma: potential role as an antiproliferative, pro-apoptotic and chemosensitizing agent. Br J Pharmacol. (2011) 163:283–98. 10.1111/j.1476-5381.2010.01187.x 21198544 PMC3087132

[64] Turkson J Jove R. STAT proteins: novel molecular targets for cancer drug discovery. Oncogene (2000) 19:6613. 10.1038/sj.onc.1204086 11426647

[65] Li F Shanmugam MK Chen L Chatterjee S Basha J Kumar AP Garcinol, a polyisoprenylated benzophenone modulates multiple pro-inflammatory signaling cascades leading to suppression of growth and survival of head and neck carcinoma. Cancer Prev Res. (2013) 6:843–54. 10.1158/1940-6207.CAPR-13-0070 23803415

[66] Silva CM. Role of STATs as downstream signal transducers in Src family kinase-mediated tumorigenesis. Oncogene (2004) 23:8017. 10.1038/sj.onc.1208159 15489919

[67] Yamashita H Xu J Erwin RA Farrar WL Kirken RA Rui H. Differential control of the phosphorylation state of proline-juxtaposed serine residues Ser725 of Stat5a and Ser730 of Stat5b in prolactin-sensitive cells. J Biol Chem. (1998) 273:30218–24. 9804779 10.1074/jbc.273.46.30218

[68] Wang R-A Vadlamudi RK Bagheri-Yarmand R Beuvink I Hynes NE Kumar RJTJocb. Essential functions of p21-activated kinase 1 in morphogenesis and differentiation of mammary glands. J Cell Biol. (2003) 161:583–92. 10.1083/jcb.200212066 12732616 PMC2172951

[69] Park S-H Yamashita H Rui H Waxman DJ. Serine phosphorylation of GH-activated signal transducer and activator of transcription 5a (STAT5a) and STAT5b: impact on STAT5 transcriptional activity. Mol Endocrinol. (2001) 15:2157–71. 10.1210/mend.15.12.0746 11731617

[70] Shanmugam MK Rajendran P Li F Nema T Vali S Abbasi T. Ursolic acid inhibits multiple cell survival pathways leading to suppression of growth of prostate cancer xenograft in nude mice. J Mol Med. (2011) 89:713. 10.1007/s00109-011-0746-2 21465181

[71] Rajendran P Ong TH Chen L Li F Shanmugam M Vali S. Suppression of signal transducer and activator of transcription 3 activation by butein inhibits growth of human hepatocellular carcinoma *in vivo*. Clin Cancer Res. (2011) 17:1425–39. 10.1158/1078-0432.CCR-10-1123 21131551

[72] Xiong H Su W-Y Liang Q-C Zhang Z-G Chen H-M Du W. Inhibition of STAT5 induces G1 cell cycle arrest and reduces tumor cell invasion in human colorectal cancer cells. Lab Invest. (2009) 89:717–25. 10.1038/labinvest.2009.11 19290007

[73] Coffer PJ Koenderman L de Groot RP. The role of STATs in myeloid differentiation and leukemia. Oncogene (2000) 19:2511. 10.1038/sj.onc.1203479 10851050

[74] Ito Y. Oncogenic potential of the RUNX gene family: ‘overview’. Oncogene (2004) 23:4198. 10.1038/sj.onc.1207755 15156173

[75] Cameron ER Neil JC. The RUNX genes: lineage-specific oncogenes and tumor suppressors. Oncogene (2004) 23:4308. 10.1038/sj.onc.1207130 15156187

[76] Osato M. Point mutations in the RUNX1/AML1 gene: another actor in RUNX leukemia. Oncogene (2004) 23:4284. 10.1038/sj.onc.1207779 15156185

[77] Pratap J Lian JB Stein GS. Metastatic bone disease: role of transcription factors and future targets. Bone (2011) 48:30–6. 10.1016/j.bone.2010.05.035 20561908 PMC2958222

[78] Thomas DM Johnson SA Sims NA Trivett MK Slavin JL Rubin BP. Terminal osteoblast differentiation, mediated by runx2 and p27KIP1, is disrupted in osteosarcoma. J Cell Biol. (2004) 167:925–34. 10.1083/jcb.200409187 15583032 PMC2172443

[79] Ito K Liu Q Salto-Tellez M Yano T Tada K Ida H. RUNX3, a novel tumor suppressor, is frequently inactivated in gastric cancer by protein mislocalization. Cancer Res. (2005) 65:7743–50. 10.1158/0008-5472.CAN-05-0743 16140942

[80] Ulloa L Doody J Massagué J. Inhibition of transforming growth factor-β/SMAD signalling by the interferon-γ/STAT pathway. Nature (1999) 397:710. 10.1038/17826 10067896

[81] Li Q-L Ito K Sakakura C Fukamachi H Inoue K-I Chi X-Z. Causal relationship between the loss of RUNX3 expression and gastric cancer. Cell (2002) 109:113–24. 10.1016/S0092-8674(02)00690-6 11955451

[82] Yano T Ito K Fukamachi H Chi X-Z Wee H-J Inoue K-i The RUNX3 tumor suppressor upregulates bim in gastric epithelial cells undergoing transforming growth factorβ-induced apoptosis. Mol Cell Biol. (2006) 26:4474–88. 10.1128/MCB.01926-05 16738314 PMC1489128

[83] Zheng J Mei Y Xiang P Zhai G Zhao N Xu C. DNA methylation affects metastasis of renal cancer and is associated with TGF-β/RUNX3 inhibition. Cancer Cell Int. (2018) 18:56. 10.1186/s12935-018-0554-7 29651226 PMC5894227

[84] Nakashima K Yanagisawa M Arakawa H Kimura N Hisatsune T Kawabata M. Synergistic signaling in fetal brain by STAT3-Smad1 complex bridged by p300. Science (1999) 284:479–82. 10.1126/science.284.5413.479 10205054

[85] Kim S Koga T Isobe M Kern BE Yokochi T Chin YE. Stat1 functions as a cytoplasmic attenuator of Runx2 in the transcriptional program of osteoblast differentiation. Genes Dev. (2003) 17:1979–91. 10.1101/gad.1119303 12923053 PMC196253

[86] Miyazono K Maeda S Imamura T. Coordinate regulation of cell growth and differentiation by TGF-β superfamily and RUNX proteins. Oncogene (2004) 23:4232. 10.1038/sj.onc.1207131 15156178

[87] Levine AJ Momand J Finlay CA. The p53 tumour suppressor gene. Nature (1991) 351:453. 10.1038/351453a0 2046748

[88] Niu G Wright KL Ma Y Wright GM Huang M Irby R. Role of Stat3 in regulating p53 expression and function. Mol Cell Biol. (2005) 25:7432–40. 10.1128/MCB.25.17.7432-7440.2005 16107692 PMC1190305

[89] Chin YE Kitagawa M Su W-CS You Z-H Iwamoto Y Fu X-Y Cell growth arrest and induction of cyclin-dependent kinase inhibitor p21WAF1/CIP1 mediated by STAT1. Science (1996) 272:719–22. 10.1126/science.272.5262.719 8614832

[90] Macleod KF Sherry N Hannon G Beach D Tokino T Kinzler K. p53-dependent and independent expression of p21 during cell growth, differentiation, and DNA damage. Genes Dev. (1995) 9:935–44. 10.1101/gad.9.8.935 7774811

[91] Townsend PA Scarabelli TM Davidson SM Knight RA Latchman DS Stephanou A. STAT-1 interacts with p53 to enhance DNA damage-induced apoptosis. J Biol Chem. (2004) 279:5811–20. 10.1074/jbc.M302637200 14602726

[92] Kaplan DH Shankaran V Dighe AS Stockert E Aguet M Old LJ. Demonstration of an interferon γ-dependent tumor surveillance system in immunocompetent mice. Proc Natl Acad Sci USA. (1998) 95:7556–61. 10.1073/pnas.95.13.7556 9636188 PMC22681

[93] Haupt Y Maya R Kazaz A Oren M. Mdm2 promotes the rapid degradation of p53. Nature (1997) 387:296. 10.1038/387296a0 9153395

[94] Baran-Marszak F Feuillard J Najjar I Le Clorennec C Béchet J-M Dusanter-Fourt I. Differential roles of STAT1α and STAT1β in fludarabine-induced cell cycle arrest and apoptosis in human B cells. Blood (2004) 104:2475–83. 10.1182/blood-2003-10-3508 15217838

[95] Youlyouz-Marfak I Gachard N Le Clorennec C Najjar I Baran-Marszak F Reminieras L. Identification of a novel p53-dependent activation pathway of STAT1 by antitumour genotoxic agents. Cell Death Differ. (2008) 15:376. 10.1038/sj.cdd.4402270 17992189

[96] Gamero AM Kotredes K Abezis A Gohimukkula SR Afanassiev A Cremers T p53 inactivation and STAT2 cooperate to enhance migration and metastasis of colon tumor cells. AACR (2018) 78:Abstract nr 3157. 10.1158/1538-7445.AM2018-3157

[97] Mantovani A Allavena P Sica A Balkwill F. Cancer-related inflammation. Nature (2008) 454:436. 10.1038/nature07205 18650914

[98] Karin M Greten FR. NF-κB: linking inflammation and immunity to cancer development and progression. Nat Rev Immunol. (2005) 5:749. 10.1038/nri1703 16175180

[99] Baud V Karin M. Is NF-κB a good target for cancer therapy? Hopes and pitfalls. Nature Rev Drug Discov. (2009) 8:33. 10.1038/nrd2781 19116625 PMC2729321

[100] Bollrath J Phesse TJ von Burstin VA Putoczki T Bennecke M Bateman T. gp130-mediated Stat3 activation in enterocytes regulates cell survival and cell-cycle progression during colitis-associated tumorigenesis. Cancer Cell (2009) 15:91–102. 10.1016/j.ccr.2009.01.002 19185844

[101] Grivennikov S Karin E Terzic J Mucida D Yu G-Y Vallabhapurapu S. IL-6 and Stat3 are required for survival of intestinal epithelial cells and development of colitis-associated cancer. Cancer Cell (2009) 15:103–13. 10.1016/j.ccr.2009.01.001 19185845 PMC2667107

[102] Lee H Herrmann A Deng J-H Kujawski M Niu G Li Z. Persistently activated Stat3 maintains constitutive NF-κB activity in tumors. Cancer Cell (2009) 15:283–93. 10.1016/j.ccr.2009.02.015 19345327 PMC2777654

[103] Yang J Liao X Agarwal MK Barnes L Auron PE Stark GR. Unphosphorylated STAT3 accumulates in response to IL-6 and activates transcription by binding to NFκB. Genes Dev. (2007) 21:1396–408. 10.1101/gad.1553707 17510282 PMC1877751

[104] Bromberg JF Wrzeszczynska MH Devgan G Zhao Y Pestell RG Albanese C. Stat3 as an oncogene. Cell (1999) 98:295–303. 10.1016/S0092-8674(00)81959-5 10458605

[105] Yu H Kortylewski M Pardoll D Tumour immunology: crosstalk between cancer and immune cells: role of STAT3 in the tumour microenvironment. Nat Rev Immunol. (2007) 7:41 10.1038/nri1995 17186030

[106] Basseres D Baldwin A. Nuclear factor-κB and inhibitor of κB kinase pathways in oncogenic initiation and progression. Oncogene (2006) 25:6817. 10.1038/sj.onc.1209942 17072330

[107] Zhong Z Wen Z Darnell JE. Stat3: a STAT family member activated by tyrosine phosphorylation in response to epidermal growth factor and interleukin-6. Science (1994) 264:95–8. 10.1126/science.8140422 8140422

[108] Yu H Jove R. The STATs of cancer—new molecular targets come of age. Nat Rev Cancer (2004) 4:97. 10.1038/nrc1275 14964307

[109] Ogura H Murakami M Okuyama Y Tsuruoka M Kitabayashi C Kanamoto M. Interleukin-17 promotes autoimmunity by triggering a positive-feedback loop via interleukin-6 induction. Immunity (2008) 29:628–36. 10.1016/j.immuni.2008.07.018 18848474

[110] Pandurangan AK Esa NM. Signal transducer and activator of transcription 3-a promising target in colitis-associated cancer. Asian Pac J Cancer Prev. (2014) 15:551–60. 10.7314/apjcp.2014.15.2.551 24568457

[111] Murone M Vaslin Chessex A Attinger A Ramachandra R Shetty SJ Daginakatte G. Debio 0617B inhibits growth of STAT3-driven solid tumors through combined inhibition of JAK, SRC, and class III/V receptor tyrosine kinases. Mol Cancer Ther. (2016) 15:2334–43. 10.1158/1535-7163.MCT-15-0974 27439479

[112] Wu P Nielsen TE Clausen MH. FDA-approved small-molecule kinase inhibitors. Trends Pharmacol Sci. (2015) 36:422–39. 10.1016/j.tips.2015.04.005 25975227

[113] Pei H He L Shao M Yang Z Ran Y Li D. Discovery of a highly selective JAK3 inhibitor for the treatment of rheumatoid arthritis. Sci Rep. (2018) 8:5273. 10.1038/s41598-018-23569-y 29588471 PMC5869712

[114] Ghoreschi K Laurence A O'Shea JJ. Janus kinases in immune cell signaling. Immunol Rev. (2009) 228:273–87. 10.1111/j.1600-065X.2008.00754.x 19290934 PMC2782696

[115] Gehringer M Forster M Pfaffenrot E Bauer SM Laufer SA. Novel Hinge-binding motifs for janus kinase 3 inhibitors: a comprehensive structure–activity relationship study on tofacitinib bioisosteres. ChemMedChem (2014) 9:2516–27. 10.1002/cmdc.201402252 25139757

[116] Winthrop KL The emerging safety profile of JAK inhibitors in rheumatic disease. Nat Rev Rheumatol. (2017) 13:234–43. 10.1038/nrrheum.2017.23 28250461

[117] Lavecchia A Di Giovanni C Novellino E. STAT-3 inhibitors: state of the art and new horizons for cancer treatment. Curr Med Chem. (2011) 18:2359–75. 10.2174/092986711795843218 21568920

[118] Buettner R Mora LB Jove R. Activated STAT signaling in human tumors provides novel molecular targets for therapeutic intervention. Clin Cancer Res. (2002) 8:945–54. 11948098

[119] Furqan M Akinleye A Mukhi N Mittal V Chen Y Liu DJJoh. STAT inhibitors for cancer therapy. J Hematol Oncol. (2013) 6:90. 10.1186/1756-8722-6-90 24308725 PMC4029528

[120] Wingelhofer B Maurer B Heyes EC Cumaraswamy AA Berger-Becvar A de Araujo ED. Pharmacologic inhibition of STAT5 in acute myeloid leukemia. Leukemia (2018) 32:1135–46. 10.1038/s41375-017-0005-9 29472718 PMC5940656

[121] Nelson EA Walker SR Weisberg E Bar-Natan M Barrett R Gashin LB. The STAT5 inhibitor pimozide decreases survival of chronic myelogenous leukemia cells resistant to kinase inhibitors. Blood (2011) 117:3421–9. 10.1182/blood-2009-11-255232 21233313 PMC3069678

[122] Arora L Kumar A Arfuso F Chng W Sethi G. The role of signal transducer and activator of transcription 3 (STAT3) and its targeted inhibition in hematological malignancies. Cancers (Basel) (2018) 10:327. 10.3390/cancers10090327 30217007 PMC6162647

[123] Shin D-S Kim H-N Shin KD Yoon YJ Kim S-J Han DC. Cryptotanshinone inhibits constitutive signal transducer and activator of transcription 3 function through blocking the dimerization in DU145 prostate cancer cells. Cancer Res. (2009) 69:193–202. 10.1158/0008-5472.CAN-08-2575 19118003

[124] Bhutani M Pathak AK Nair AS Kunnumakkara AB Guha S Sethi G. Capsaicin is a novel blocker of constitutive and interleukin-6–inducible STAT3 activation. Clin Cancer Res. (2007) 13:3024–32. 10.1158/1078-0432.CCR-06-2575 17505005

[125] Bharti AC Donato N Aggarwal BB. Curcumin (diferuloylmethane) inhibits constitutive and IL-6-inducible STAT3 phosphorylation in human multiple myeloma cells. J Immunol. (2003) 171:3863–71. 10.4049/jimmunol.171.7.3863 14500688

[126] Oi T Asanuma K Matsumine A Matsubara T Nakamura T Iino T. STAT3 inhibitor, cucurbitacin I, is a novel therapeutic agent for osteosarcoma. Int J Oncol. (2016) 49:2275–84. 10.3892/ijo.2016.3757 27840900 PMC5117998

[127] Amit-Vazina M Shishodia S Harris D Van Q Wang M Weber D. Atiprimod blocks STAT3 phosphorylation and induces apoptosis in multiple myeloma cells. Br J Cancer (2005) 93:70–80. 10.1038/sj.bjc.6602637 15970928 PMC2361492

[128] Pinz S Unser S Rascle A. The natural chemopreventive agent sulforaphane inhibits STAT5 activity. PLoS ONE (2014) 9:e99391. 10.1371/journal.pone.0099391 24910998 PMC4051870

[129] Alshamsan A Hamdy S Samuel J El-Kadi AO Lavasanifar A Uludag H. The induction of tumor apoptosis in B16 melanoma following STAT3 siRNA delivery with a lipid-substituted polyethylenimine. Biomaterials (2010) 31:1420–8. 10.1016/j.biomaterials.2009.11.003 19913908

[130] Turkson J. STAT proteins as novel targets for cancer drug discovery. Expert Opin Ther Targets (2004) 8:409–22. 10.1517/14728222.8.5.409 15469392

[131] Anastasov N Klier M Koch I Angermeier D Höfler H Fend F. Efficient shRNA delivery into B and T lymphoma cells using lentiviral vector-mediated transfer. J Hematopathol. (2009) 2:9–19. 10.1007/s12308-008-0020-x 19669218 PMC2713496

[132] Peer D Lieberman J. Special delivery: targeted therapy with small RNAs. Gene Ther. (2011) 18:1127. 10.1038/gt.2011.56 21490679

[133] Weinstein S Toker IA Emmanuel R Ramishetti S Hazan-Halevy I Rosenblum D. Harnessing RNAi-based nanomedicines for therapeutic gene silencing in B-cell malignancies. Proc Natl Acad Sci USA. (2016) 113:E16–22. 10.1073/pnas.1519273113 26699502 PMC4711827

[134] de Fougerolles A Vornlocher H-P Maraganore J Lieberman J. Interfering with disease: a progress report on siRNA-based therapeutics. Nat Rev Drug Discov. (2007) 6:443–53. 10.1038/nrd2310 17541417 PMC7098199

[135] Paddison PJ Caudy AA Bernstein E Hannon GJ Conklin DS. Short hairpin RNAs (shRNAs) induce sequence-specific silencing in mammalian cells. Genes Dev. (2002) 16:948–58. 10.1101/gad.981002 11959843 PMC152352

[136] Konnikova L Kotecki M Kruger MM Cochran BH. Knockdown of STAT3 expression by RNAi induces apoptosis in astrocytoma cells. BMC Cancer (2003) 3:23. 10.1186/1471-2407-3-23 13678425 PMC212316

[137] Konnikova L Simeone MC Kruger MM Kotecki M Cochran BH. Signal transducer and activator of transcription 3 (STAT3) regulates human telomerase reverse transcriptase (hTERT) expression in human cancer and primary cells. Cancer Res. (2005) 65:6516–20. 10.1158/0008-5472.CAN-05-0924 16061629

[138] Cao S Wang C Zheng Q Qiao Y Xu K Jiang T. STAT5 regulates glioma cell invasion by pathways dependent and independent of STAT5 DNA binding. Neurosci Lett. (2011) 487:228–33. 10.1016/j.neulet.2010.10.028 20969921

[139] Gu L Vogiatzi P Puhr M Dagvadorj A Lutz J Ryder A. Stat5 promotes metastatic behavior of human prostate cancer cells *in vitro* and *in vivo*. Endocr Relat Cancer (2010) 17:481–93. 10.1677/ERC-09-0328 20233708 PMC6260789

[140] Choi JK Kim KH Park H Park SR Choi BH. Granulocyte macrophage-colony stimulating factor shows anti-apoptotic activity in neural progenitor cells via JAK/STAT5-Bcl-2 pathway. Apoptosis (2011) 16:127–34. 10.1007/s10495-010-0552-2 21052840

[141] Gao LF Xu DQ Wen LJ Zhang XY Shao YT Zhao XJ. Inhibition of STAT3 expression by siRNA suppresses growth and induces apoptosis in laryngeal cancer cells. Acta Pharmacol Sin. (2005) 26:377–83. 10.1111/j.1745-7254.2005.00053.x 15715937

[142] Jin D Zang W Wang T Li M Wan J Zhao G The effect of STAT5 silenced by siRNA on proliferation, apoptosis and invasion of esophageal carcinoma cell line EC9706. Chinese German J Clin Oncol. (2010) 9:692–6. 10.1007/s10330-010-0717-z

[143] Kunigal S Lakka SS Sodadasu PK Estes N Rao JS. Stat3-siRNA induces Fas-mediated apoptosis *in vitro* and *in vivo* in breast cancer. Int J Oncol. (2009) 34:1209–20. 10.3892/ijo_00000249 19360334 PMC2668130

[144] Xiong H Hong J Du W Lin Y-w Ren L-l Wang Y-c. Roles of STAT3 and ZEB1 proteins in E-cadherin down-regulation and human colorectal cancer epithelial-mesenchymal transition. J Biol Chem. (2012) 287:5819–32. 10.1074/jbc.M111.295964 22205702 PMC3285352

[145] Xu X Dong Z Li Y Yang Y Yuan Z Qu X. The upregulation of signal transducer and activator of transcription 5-dependent microRNA-182 and microRNA-96 promotes ovarian cancer cell proliferation by targeting forkhead box O3 upon leptin stimulation. Int J Biochem Cell Biol. (2013) 45:536–45. 10.1016/j.biocel.2012.12.010 23262295

[146] Zhang W Sun H Shi X Wang H Cui C Xiao F. SENP1 regulates hepatocyte growth factor-induced migration and epithelial-mesenchymal transition of hepatocellular carcinoma. Tumour Biol. (2016) 37:7741–8. 10.1007/s13277-015-4406-y 26695141

[147] Tatiparti K Sau S Kashaw SK Iyer AK. siRNA delivery strategies: a comprehensive review of recent developments. Nanomaterials (2017) 7:77. 10.3390/nano7040077 28379201 PMC5408169

[148] Guven H Konstantinidis KV Alici E Aints A Abedi-Valugerdi M Christensson B. Efficient gene transfer into primary human natural killer cells by retroviral transduction. Exp Hematol. (2005) 33:1320–8. 10.1016/j.exphem.2005.07.006 16263416

[149] Yang G Huang C Cao J Huang K-J Jiang T Qiu Z-J. Lentivirus-mediated shRNA interference targeting STAT3 inhibits human pancreatic cancer cell invasion. World J Gastroenterol. (2009) 15:3757. 10.3748/wjg.15.3757 19673016 PMC2726453

[150] Emeagi P Maenhout S Dang N Heirman C Thielemans K Breckpot K. Downregulation of Stat3 in melanoma: reprogramming the immune microenvironment as an anticancer therapeutic strategy. Gene Ther. (2013) 20:1085. 10.1038/gt.2013.35 23804077

[151] Wang X Liu P Liu H Yang W Liu Z Zhuo Z. Delivery of interferons and siRNA targeting STAT3 using lentiviral vectors suppresses the growth of murine melanoma. Cancer Gene Ther. (2012) 19:822. 10.1038/cgt.2012.65 23018621

[152] Cao S Yan Y Zhang X Zhang K Liu C Zhao G. EGF stimulates cyclooxygenase-2 expression through the STAT5 signaling pathway in human lung adenocarcinoma A549 cells. Int J Oncol. (2011) 39:383–91. 10.3892/ijo.2011.1053 21617857

[153] Zhang S Zhao B Jiang H Wang B Ma B. Cationic lipids and polymers mediated vectors for delivery of siRNA. J Control Release (2007) 123:1–10. 10.1016/j.jconrel.2007.07.016 17716771

[154] Zhao Y Zheng C Zhang L Chen Y Ye Y Zhao M. Knockdown of STAT3 expression in SKOV3 cells by biodegradable siRNA–PLGA/CSO conjugate micelles. Colloids Surf B Biointerfaces (2015) 127:155–63. 10.1016/j.colsurfb.2015.01.034 25677339

[155] Hou KK Pan H Lanza GM Wickline SA. Melittin derived peptides for nanoparticle based siRNA transfection. Biomaterials (2013) 34:3110–9. 10.1016/j.biomaterials.2013.01.037 23380356 PMC3578292

[156] Soenen SJ Brisson AR De Cuyper M. Addressing the problem of cationic lipid-mediated toxicity: the magnetoliposome model. Biomaterials (2009) 30:3691–701. 10.1016/j.biomaterials.2009.03.040 19371948

[157] Ballarín-González B Howard KA. Polycation-based nanoparticle delivery of RNAi therapeutics: adverse effects and solutions. Adv Drug Deliv Rev. (2012) 64:1717–29. 10.1016/j.addr.2012.07.004 22800620

[158] Forti E Kryukov O Elovic E Goldshtein M Korin E Margolis G. A bridge to silencing: Co-assembling anionic nanoparticles of siRNA and hyaluronan sulfate via calcium ion bridges. J Control Release (2016) 232:215–27. 10.1016/j.jconrel.2016.04.033 27117458

[159] Lv H Zhang S Wang B Cui S Yan J. Toxicity of cationic lipids and cationic polymers in gene delivery. J Control Release (2006) 114:100–9. 10.1016/j.jconrel.2006.04.014 16831482

[160] Alshamsan A Haddadi A Incani V Samuel J Lavasanifar A Uludag H. Formulation and delivery of siRNA by oleic acid and stearic acid modified polyethylenimine. Mol Pharma. (2008) 6:121–33. 10.1021/mp8000815 19053537

[161] Das J Das S Paul A Samadder A Bhattacharyya SS Khuda-Bukhsh AR. Assessment of drug delivery and anticancer potentials of nanoparticles-loaded siRNA targeting STAT3 in lung cancer, *in vitro* and *in vivo*. Toxicol Lett. (2014) 225:454–66. 10.1016/j.toxlet.2014.01.009 24440344

[162] Su W-P Cheng F-Y Shieh D-B Yeh C-S Su W-C. PLGA nanoparticles codeliver paclitaxel and Stat3 siRNA to overcome cellular resistance in lung cancer cells. Int J Nanomed. (2012) 7:4269. 10.2147/IJN.S33666 22904633 PMC3418083

[163] Yin D Li Y Lin H Guo B Du Y Li X. Functional graphene oxide as a plasmid-based Stat3 siRNA carrier inhibits mouse malignant melanoma growth *in vivo*. Nanotechnology (2013) 24:105102. 10.1088/0957-4484/24/10/105102 23425941

[164] Yuan X Naguib S Wu Z. Recent advances of siRNA delivery by nanoparticles. Expert Opin Drug Deliv. (2011) 8:521–36. 10.1517/17425247.2011.559223 21413903

[165] Heo MB Lim YT. Programmed nanoparticles for combined immunomodulation, antigen presentation and tracking of immunotherapeutic cells. Biomaterials (2014) 35:590–600. 10.1016/j.biomaterials.2013.10.009 24125775

[166] Molavi O Mahmud A Hamdy S Hung RW Lai R Samuel J. Development of a poly (d, l-lactic-co-glycolic acid) nanoparticle formulation of STAT3 inhibitor JSI-124: implication for cancer immunotherapy. Mol Pharma. (2010) 7:364–74. 10.1021/mp900145g 20030320

[167] Labala S Jose A Chawla SR Khan MS Bhatnagar S Kulkarni OP. Effective melanoma cancer suppression by iontophoretic co-delivery of STAT3 siRNA and imatinib using gold nanoparticles. Int J Pharma. (2017) 525:407–17. 10.1016/j.ijpharm.2017.03.087 28373100

[168] Labala S Jose A Venuganti VVK. Transcutaneous iontophoretic delivery of STAT3 siRNA using layer-by-layer chitosan coated gold nanoparticles to treat melanoma. Colloids Surf B Biointerfaces (2016) 146:188–97. 10.1016/j.colsurfb.2016.05.076 27318964

[169] Tan WB Zhang Y. Surface modification of gold and quantum dot nanoparticles with chitosan for bioapplications. J Biomed Mater Res A (2005) 75:56–62. 10.1002/jbm.a.30410 16086419

[170] Liang Z-W Guo B-F Li Y Li X-J Li X Zhao L-J. Plasmid-based Stat3 siRNA delivered by hydroxyapatite nanoparticles suppresses mouse prostate tumour growth *in vivo*. Asian J Androl. (2011) 13:481. 10.1038/aja.2010.167 21297658 PMC3739337

[171] Shi K Fang Y Gao S Yang D Bi H Xue J. Inorganic kernel-Supported asymmetric hybrid vesicles for targeting delivery of STAT3-decoy oligonucleotides to overcome anti-HER2 therapeutic resistance of BT474R. J Control Release (2018) 279:53–68. 10.1016/j.jconrel.2018.04.023 29655990

[172] Luo Z Wang C Yi H Li P Pan H Liu L. Nanovaccine loaded with poly I: C and STAT3 siRNA robustly elicits anti-tumor immune responses through modulating tumor-associated dendritic cells *in vivo*. Biomaterials (2015) 38:50–60. 10.1016/j.biomaterials.2014.10.050 25457983

[173] Ou W Thapa RK Jiang L Soe ZC Gautam M Chang J-H. Regulatory T cell-targeted hybrid nanoparticles combined with immuno-checkpoint blockage for cancer immunotherapy. J Control Release (2018) 281:84–96. 10.1016/j.jconrel.2018.05.018 29777794

[174] Larmonier N Janikashvili N LaCasse CJ Larmonier CB Cantrell J Situ E. Imatinib mesylate inhibits CD4^+^ CD25^+^ regulatory T cell activity and enhances active immunotherapy against BCR-ABL^−^ tumors. J Immunol. (2008) 181:6955–63. 10.4049/jimmunol.181.10.6955 18981115 PMC2579962

[175] Kim SK Huang LJ. Nanoparticle delivery of a peptide targeting EGFR signaling. J Control Release (2012) 157:279–86. 10.1016/j.jconrel.2011.08.014 21871507 PMC3229664

[176] Liu L Liu YL Liu GX Chen X Yang K Yang YX. Curcumin ameliorates dextran sulfate sodium-induced experimental colitis by blocking STAT3 signaling pathway. Int Immunopharmacol. (2013) 17:314–20. 10.1016/j.intimp.2013.06.020 23856612

[177] Rathore R Jain JP Srivastava A Jachak S Kumar N. Simultaneous determination of hydrazinocurcumin and phenol red in samples from rat intestinal permeability studies: HPLC method development and validation. J Pharma Biomed Anal. (2008) 46:374–80. 10.1016/j.jpba.2007.09.019 17988818

[178] Zhang X Tian W Cai X Wang X Dang W Tang H. Hydrazinocurcumin encapsuled nanoparticles “re-educate” tumor-associated macrophages and exhibit anti-tumor effects on breast cancer following STAT3 suppression. PLoS ONE (2013) 8:e65896. 10.1371/journal.pone.0065896 23825527 PMC3692525

[179] Liao D Liu Z Wrasidlo W Chen T Luo Y Xiang R. Synthetic enzyme inhibitor: a novel targeting ligand for nanotherapeutic drug delivery inhibiting tumor growth without systemic toxicity. Nanomedicine (2011) 7:665–73. 10.1016/j.nano.2011.03.001 21419870

[180] Guorgui J Wang R Mattheolabakis G Mackenzie GG. Curcumin formulated in solid lipid nanoparticles has enhanced efficacy in Hodgkin's lymphoma in mice. Arch Biochem Biophys. (2018) 648:12–9. 10.1016/j.abb.2018.04.012 29679536

[181] Ma Y Zhang X Xu X Shen L Yao Y Yang Z. STAT3 decoy oligodeoxynucleotides-loaded solid lipid nanoparticles induce cell death and inhibit invasion in ovarian cancer cells. PLoS ONE (2015) 10:e0124924. 10.1371/journal.pone.0124924 25923701 PMC4414561

[182] Kotmakçi M Çetintaş VB Kantarci AG. Preparation and characterization of lipid nanoparticle/pDNA complexes for STAT3 downregulation and overcoming chemotherapy resistance in lung cancer cells. Int J Pharma. (2017) 525:101–11. 10.1016/j.ijpharm.2017.04.034 28428090

[183] Xu Y Lu S. A meta-analysis of STAT3 and phospho-STAT3 expression and survival of patients with non-small-cell lung cancer. Eur J Surg Oncol. (2014) 40:311–7. 10.1016/j.ejso.2013.11.012 24332948

[184] Shahzad MM Mangala LS Han HD Lu C Bottsford-Miller J Nishimura M. Targeted delivery of small interfering RNA using reconstituted high-density lipoprotein nanoparticles. Neoplasia (2011) 13:309. 10.1593/neo.101372 21472135 PMC3071079

[185] Ruvinov E Kryukov O Forti E Korin E Goldstein M Cohen S. Calcium–siRNA nanocomplexes: what reversibility is all about. J Control Release (2015) 203:150–60. 10.1016/j.jconrel.2015.02.029 25702963

[186] Bondalapati S Ruvinov E Kryukov O Cohen S Brik A Rapid end-group modification of polysaccharides for biomaterial applications in regenerative medicine. Macromol Rapid Commun. (2014) 35:1754–62. 10.1002/marc.201400354 25220432

[187] Korin E Bejerano T Cohen S. GalNAc bio-functionalization of nanoparticles assembled by electrostatic interactions improves siRNA targeting to the liver. J Control Release (2017) 266:310–20. 10.1016/j.jconrel.2017.10.001 28987883

[188] de Almeida CE Alves LN Rocha HF Cabral-Neto JB Missailidis S. Aptamer delivery of siRNA, radiopharmaceutics and chemotherapy agents in cancer. Int J Pharma. (2017) 525:334–42. 10.1016/j.ijpharm.2017.03.086 28373101

[189] Esposito CL Nuzzo S Catuogno S Romano S de Nigris F de Franciscis V. STAT3 gene silencing by aptamer-siRNA chimera as selective therapeutic for glioblastoma. Mol Ther Nucleic Acids (2018) 10:398–411. 10.1016/j.omtn.2017.12.021 29499951 PMC5862137

[190] Akhavan D Pourzia AL Nourian AA Williams KJ Nathanson D Babic I. De-repression of PDGFRβ transcription promotes acquired resistance to EGFR tyrosine kinase inhibitors in glioblastoma patients. Cancer Discov. (2013):3:534–47. 10.1158/2159-8290.CD-12-0502 23533263 PMC3651754

[191] Najar AG Pashaei-Asl R Omidi Y Farajnia S Nourazarian AR. EGFR antisense oligonucleotides encapsulated with nanoparticles decrease EGFR, MAPK1 and STAT5 expression in a human colon cancer cell line. Asian Pac J Cancer Prev. (2013) 14:495–8. 10.7314/apjcp.2013.14.1.495 23534780

[192] Jiang X Bugno J Hu C Yang Y Herold T Qi J. Eradication of acute myeloid leukemia with FLT3 ligand-targeted miR-150 nanoparticles. Cancer Res. (2016) 76:4470–80. 10.1158/0008-5472.CAN-15-2949 27280396 PMC4970973

[193] Théry C Zitvogel L Amigorena S. Exosomes: composition, biogenesis and function. Nat Rev Immunol. (2002) 2:569–79. 10.1038/nri855 12154376

[194] Zhang H Freitas D Kim HS Fabijanic K Li Z Chen H. Identification of distinct nanoparticles and subsets of extracellular vesicles by asymmetric flow field-flow fractionation. Nat Cell Biol. (2018) 20:332–343. 10.1038/s41556-018-0040-4 29459780 PMC5931706

